# Proteomics analysis of the p.G849D variant in neurexin 2 alpha may reveal insight into Parkinson’s disease pathobiology

**DOI:** 10.3389/fnagi.2022.1002777

**Published:** 2022-11-30

**Authors:** Katelyn Cuttler, Suereta Fortuin, Amica Corda Müller-Nedebock, Maré Vlok, Ruben Cloete, Soraya Bardien

**Affiliations:** ^1^Division of Molecular Biology and Human Genetics, Faculty of Medicine and Health Sciences, Stellenbosch University, Cape Town, South Africa; ^2^Faculty of Medicine and Health Sciences, African Microbiome Institute, Stellenbosch University, Cape Town, South Africa; ^3^South African Medical Research Council/Stellenbosch University Genomics of Brain Disorders Research Unit, Cape Town, South Africa; ^4^Mass Spectrometry Unit, Central Analytical Facilities, Stellenbosch University, Cape Town, South Africa; ^5^South African Medical Research Council Bioinformatics Unit, South African National Bioinformatics Institute, University of the Western Cape, Cape Town, South Africa

**Keywords:** neurexin 2α (*NRXN2*), Parkinson’s disease, proteomics, mass spectrometry, p.G849D, synaptic translation, mitochondrial dysfunction, ribosomal functioning

## Abstract

Parkinson’s disease (PD), the fastest-growing neurological disorder globally, has a complex etiology. A previous study by our group identified the p.G849D variant in *neurexin 2* (*NRXN2*), encoding the synaptic protein, NRXN2α, as a possible causal variant of PD. Therefore, we aimed to perform functional studies using proteomics in an attempt to understand the biological pathways affected by the variant. We hypothesized that this may reveal insight into the pathobiology of PD. Wild-type and mutant NRXN2α plasmids were transfected into SH-SY5Y cells. Thereafter, total protein was extracted and prepared for mass spectrometry using a Thermo Scientific Fusion mass spectrometer equipped with a Nanospray Flex ionization source. The data were then interrogated against the UniProt *H. sapiens* database and afterward, pathway and enrichment analyses were performed using *in silico* tools. Overexpression of the wild-type protein led to the enrichment of proteins involved in neurodegenerative diseases, while overexpression of the mutant protein led to the decline of proteins involved in ribosomal functioning. Thus, we concluded that the wild-type NRXN2α may be involved in pathways related to the development of neurodegenerative disorders, and that biological processes related to the ribosome, transcription, and tRNA, specifically at the synapse, could be an important mechanism in PD. Future studies targeting translation at the synapse in PD could therefore provide further information on the pathobiology of the disease.

## Introduction

Parkinson’s disease (PD) is an incurable neurodegenerative disorder which primarily affects movement, resulting in bradykinesia, rigidity, postural instability, resting tremor, and a range of neuropsychiatric symptoms. Notably, it has been reported to be the fastest-growing neurological disorder globally (Feigin et al., 2017), affecting over 6 million people (Dorsey et al., 2018). Over the past two and a half decades, several genetic causes of PD have been identified, implicating various biological processes including mitochondrial dysfunction, toxic protein accumulation, dysfunctional vesicle recycling, and synaptic dysfunction in PD development (Panicker et al., 2021).

Recently, we reported the finding of a p.G849D variant in the neurexin 2 gene (*NRXN2*) in a South African multiplex PD family (Sebate et al., 2021). The translated protein, NRXN2α, is a synaptic regulation protein involved in processes such as calcium channel regulation, neuronal cell adhesion, and transmembrane signaling (Craig and Kang, 2007). There have been a limited number of studies on NRXN2α in disease, but a few have implicated the protein in neuronal and synaptic disorders (Missler et al., 2003; Dachtler et al., 2015). In addition, it has been shown that neurexins and their common binding partners, neuroligins, link synaptic dysfunction to cognitive disease (Südhof, 2008).

Here, we aimed to further investigate the effect of the p.G849D variant on biological pathways by using a proteomics approach in an SH-SY5Y cellular model of PD, transfected with wild-type and mutant NRXN2α plasmids. To this end, we examined the total proteome of the different treatment groups in an attempt to understand the changes in biological pathways. We hypothesized that overexpression of the wild-type NRXN2α would provide an indication of the pathways related to NRXN2α’s function, while overexpression of the mutant NRXN2α could give an indication of the method of action by which it potentially leads to neurodegeneration. Together, these findings may provide a better understanding of the function of both the wild-type and mutant NRXN2α, and its possible involvement in PD.

## Materials and methods

### Ethical considerations

Ethical approval was obtained from the Health Research Ethics Committee (Protocol numbers 2002/C059 and S20/01/005 PhD) and the Research Ethics Committee: Biological and Environmental Safety (Protocol number BEE-2021-13149). Both committees are located at Stellenbosch University, Cape Town, South Africa.

### Cell culture

SH-SY5Y cells were cultured in DMEM with high glucose (4.5 g/l) and 4 mM L-Glutamine (Lonza). In addition, the media was supplemented with 15% FBS (Gibco) and 1% penicillin/streptomycin (Sigma Aldrich). Cells were maintained at 37°C and 5% CO_2_ in a humidified incubator (ESCO Technologies).

### Plasmids

#### NRXN2α wild-type

The NRXN2α-ECFP-N1 plasmid is a kind gift from Prof. Ann Marie Craig (University of British Columbia, Canada). This plasmid expresses wild-type mouse NRXN2α-CFP and was generated as per Kang et al. (2008). The pECFP-N1 plasmid without an insert (empty vector) was a kind gift from Prof. Harald Sitte (Medical University of Vienna, Austria).

#### Site-directed mutagenesis

In order to generate the p.G849D mutant plasmid [p.G882D in our mouse model, mouse genomic position: 540693_chr19 (GRCm39)], site-directed mutagenesis was performed on the wild-type NRXN2α-ECFP-N1 plasmid using the Q5 Site-Directed Mutagenesis kit (New England Biolabs), as per the manufacturer’s instructions. More information on the primers used and PCR conditions can be found in the Supplementary material.

### Treatment groups

A total of four treatment groups were used for the analysis: (1) non-transfected cells (NT), (2) cells transfected with the wild-type plasmid (WT), (3) cells transfected with the mutant plasmid (MUT), and (4) cells transfected with the empty vector (EV). All treatments were performed in triplicate.

### Transfection

SH-SY5Y cells were grown in sterile 25 cm^3^ flasks until 70% confluent and transfected using Lipofectamine3000 (Invitrogen) as per the manufacturer’s instructions. The transfection efficiency was determined by examining the cells under an Oxion Inverso Fluo E4 fluorescent microscope (Euromex) at 100x magnification for the presence of cyan fluorescent protein (CFP).

### NRXN2α levels

Prior to proteomics analysis, NRXN2α protein levels were determined using immunofluorescent flow cytometry and measured with the Guava^®^ Muse^®^ Cell Analyzer (Luminex) to confirm overexpression of NRXN2α. Please see Supplementary material for more details.

### Proteomics analysis

#### Protein extraction and clean-up

Cells were detached using Trypsin–EDTA and centrifuged at 2739 ×*g* for 5 min to collect cell pellets. Cell pellets were stored at −80°C until required. The pellets were then thawed in 100 mM Tris buffer pH 8 containing 0.5% sodium dodecyl sulfate (SDS, Sigma), 100 mM NaCl (Sigma), 5 mM triscarboxyethyl phosphine (TCEP, Sigma), protease inhibitor cocktail (Thermo Fisher), and 2 mM EDTA (Thermo Fisher). Once thawed, the pellets were submerged in an ice-cold sonic bath for 30 s prior to vortexing for 30 s. This cycle was repeated three times and the pellets were completely dissolved. Extraction reagents were then removed using a chloroform-methanol–water liquid–liquid extraction method. More information on the protein extraction, on-bead digest and liquid chromatography performed in preparation for mass spectrometry, can be found in the Supplementary material.

#### Mass spectrometry

Mass spectrometry was performed by Stellenbosch University’s Central Analytical Facilities (CAF) using a Thermo Scientific Fusion mass spectrometer equipped with a Nanospray Flex ionization source. The sample was introduced through a stainless-steel emitter. Data were collected in positive mode with spray voltage set to 1.8 kV and ion transfer capillary set to 280°C. Spectra were internally calibrated using polysiloxane ions at m/z = 445.12003 and 371.10024. MS1 scans were performed using the Orbitrap detector set at 120,000 resolution over the scan range 350–1,650 with automatic gain control (AGC) target at 3 E5 and maximum injection time of 40 milliseconds. Data were acquired in profile mode.

MS2 acquisitions were performed using monoisotopic precursor selection for ion with charges +2 − +7 with error tolerance set to ± 10 ppm. Precursor ions were excluded from fragmentation once for a period of 60 s. Precursor ions were selected for fragmentation in higher-energy C-trap dissociation (HCD) mode using the quadrupole mass analyzer with HCD energy set to 32.5%. Fragment ions were detected in the Orbitrap mass analyzer set to 30,000 resolution. The AGC target was set to 5E4 and the maximum injection time to 80 milliseconds. The data were acquired in centroid mode.

#### Data analysis

The raw files generated by the mass spectrometer were imported into Proteome Discoverer v1.4 (Thermo Fisher) and processed using the SequestHT algorithm. Database interrogation was performed against the UniProt *H. Sapiens* database concatenated with the cRAP contaminant protein database.^1^ Semi-tryptic cleavage with 2 missed cleavages was allowed for. Precursor mass tolerance was set to 10 ppm and fragment mass tolerance set to 0.02 Da. Deamidation (NQ) and oxidation (M) were allowed as dynamic modifications and thiomethyl of C as static modification. Peptide validation was performed using the Target-Decoy PSM validator node. The results files were imported into Scaffold 1.4.4 (Searle, 2010) and identified peptides validated with X!Tandem and the Peptide and Protein Prophet algorithms included in Scaffold. Quantitation was performed by Scaffold after one-way ANOVA and Student’s *t*-test were performed.

### Pathway analysis and enrichment analysis

First, the data for the separate treatment groups were combined into a Venn diagram using Venny 2.1^2^ (Oliveros, 2015) to identify proteins unique to each treatment group. Thereafter, in order to identify differentially abundant proteins, the data were compared as follows: empty vector transfected cells vs non-transfected cells (EV vs NT), wild-type transfected cells vs non-transfected cells (WT vs NT), mutant transfected cells vs non-transfected cells (MUT vs NT), and mutant transfected cells vs wild-type transfected cells (MUT vs WT). Functional information for unique and differentially abundant proteins was obtained from UniProt^3^ (The UniProt Consortium, 2021). Pathway analysis was conducted using the KEGG^4^ (Kanehisa et al., 2021) and STRING^5^ (Szklarczyk et al., 2019) databases. Each protein set was then uploaded to WebGestalt^6^ (Liao et al., 2019) for enrichment analysis as per the default parameters.

## Results

### Overexpression of NRXN2α

Transfection efficiency, determined by evaluating CFP microscopically, was 68% for the wild-type construct, 65% for the mutant construct, and 67% for the empty vector construct. In addition, overexpression of NRXN2α was confirmed using the Guava^®^ Muse^®^ Cell Analyzer (Luminex). There was a 26 and 21% increase in NRXN2α levels in the wild-type and mutant samples, respectively, with no change in the empty vector sample when compared to non-transfected cells (Supplementary Figure S1).

### Total proteins identified

**Figure 1:**
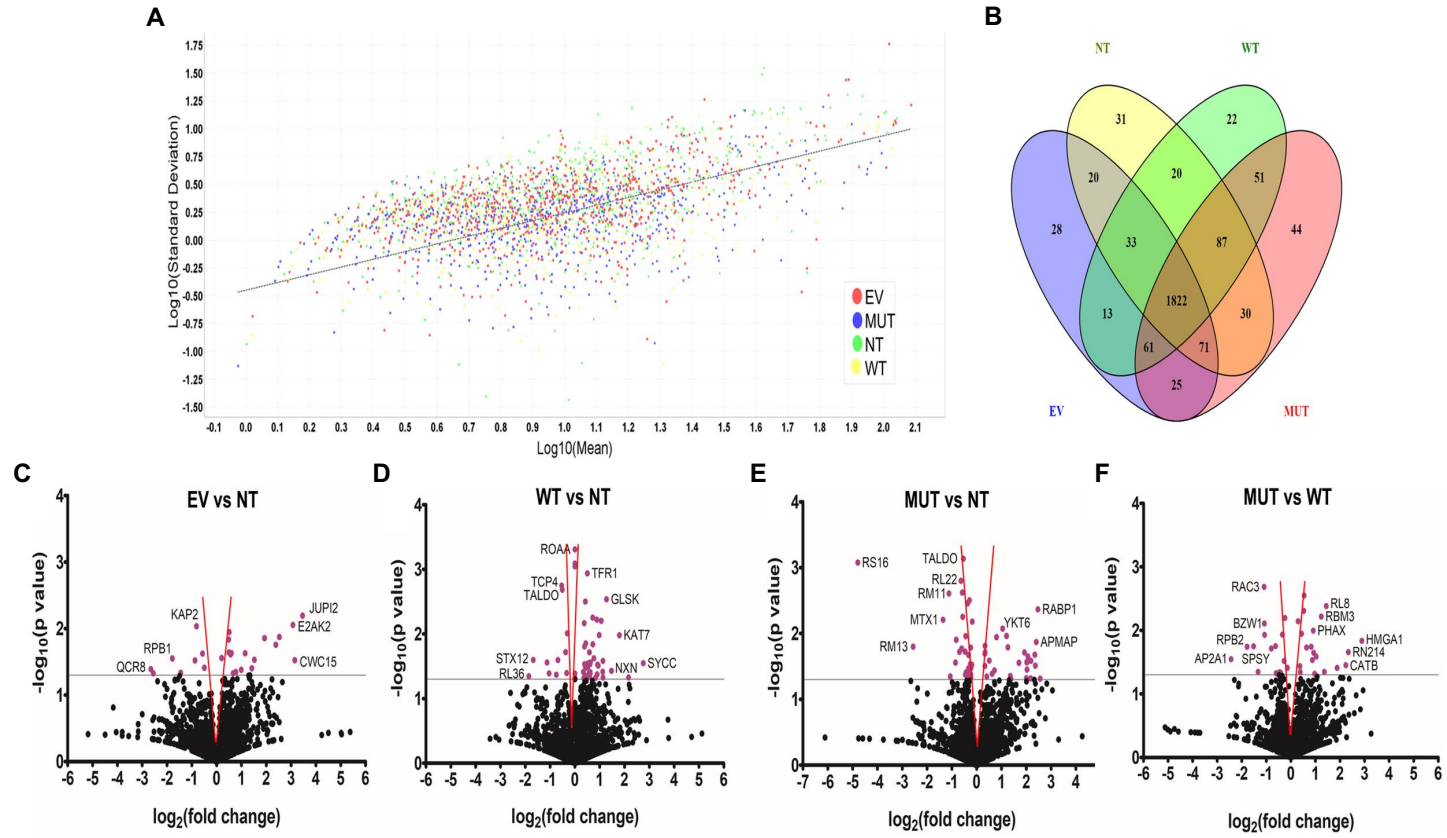
Unique and differentially abundant proteins identified in this experiment. **(A)** Scatterplot of the standard deviation (log_10_) vs mean (log_10_) shows good clustering of all samples along the linear regression line. Graph generated by Scaffold 1.4.4. **(B)** Venn diagram of the total proteins identified in each treatment group shows the distribution of shared proteins and numbers of proteins unique to each group. Diagram generated with Venny 2.1 (https://bioinfogp.cnb.csic.es/tools/venny; Oliveros, 2015). Volcano plots showing differentially abundant proteins when comparing EV vs NT **(C)**, WT vs NT **(D)**, MUT vs NT **(E)**, and MUT vs WT **(F)**. The gray line indicates the significance threshold. Significant proteins (*p* ≤ 0.05, Students *t*-test) are colored purple. Where possible, individual proteins have been labelled. Proteins found within the red funnel may become statistically insignificant with increased sample sizes. Graphs generated with GraphPad Prism^®^ 5.02. Abbreviations: EV: empty vector transfected cells, MUT: mutant transfected cells; NT: non-transfected cells; WT: wild-type transfected cells.

The total ion chromatograms in Supplementary Figure S2 show the successful digestion of peptides in each sample as well as their protein profile. Quantitation and regression analysis performed in Scaffold showed that most data points clustered within one standard deviation from the mean and that all the sample sets showed the same grouping (Figure 1A), indicating a successful experiment. The total number of proteins detected was 2,667, 2,630, 2,691, and 2,646 for the non-transfected cells (NT), wild-type transfected cells (WT), mutant transfected cells (MUT), and empty vector transfected cells (EV), respectively. Since all treatments were performed in triplicate, a protein had to be present in a minimum of 2 replicates in order to be considered an identified protein.

### Unique proteins in each group

The Venn diagram in Figure 1B shows that 1822 proteins were shared by all four treatment groups. NT cells had 31 unique proteins, while WT, MUT, and EV cells had 22, 44, and 28 unique proteins, respectively. Functional information for all of these proteins can be found in Supplementary Tables S1–S4. Each protein set was then uploaded to STRING^7^ (Szklarczyk et al., 2019) for pathway analysis.

Enrichment terms obtained from STRING for the unique proteins in each treatment group are shown in Table 1. There were no enriched terms for the EV cells, so they were excluded from the table. The NT cells showed enrichment terms for “compound binding.” “Acetylation” was the only term enriched for in the WT cells. STRING analysis of the proteins involved in acetylation shows that they are not predicted to interact with each other and do not form part of the same networks. Therefore, enrichment of “acetylation” in these cells is likely to be a chance finding, showing that the cells are undergoing modification upon transfection with the WT plasmid. However, since acetylation of proteins is also potentially implicated in neurodegenerative disorders, such as PD (Yakhine-Diop et al., 2019), it could also be an important mechanism of action for the WT NRXN2α. “Metabolic processes” were enriched in the MUT cells, which is also possibly a result of introducing the MUT NRXN2α into the cells. Interestingly, terms related to RNA processes were also enriched in the MUT cells. These cells contain several unique proteins which are involved in RNA metabolism and processing, thus showing that there may be changes in transcription in these cells. Therefore, it is possible that the MUT protein is somehow disrupting RNA processing.

**Table 1:** Enrichment terms obtained from the STRING online tool for the unique proteins in each treatment group.

NT (no. of proteins)	WT (no. of proteins)	MUT (no. of proteins)
Heterocyclic compound binding (24)	Acetylation (12)	Cellular metabolic process (31)
Organic compound binding (24)		Macromolecule metabolic process (28)
		RNA processing (12)
		Metabolism of RNA (9)

STRING: https://string-db.org (Szklarczyk et al., 2019). NT: non-transfected cells; MUT: mutant transfected cells; and WT: wild-type transfected cells.

### Differentially abundant proteins between groups

For downstream analyses, all proteins identified in the EV cells were then compared to those in the NT cells as a control since we speculate that the vector backbone should not cause significant changes to the cellular proteome. The proteins in the WT cells and MUT cells were then each compared to the NT to better understand their individual contributions to the proteome. Finally, the main analysis involved comparison of the MUT cells to the WT cells in an attempt to understand the effect of the p.G849D variant. Table 2 shows the number of differentially abundant proteins found for each comparison group, divided into those that are less abundant and those that are more abundant. Volcano plots representing the differentially abundant proteins in each analysis are shown in Figures 1C–F. Functional information for all of these proteins can be found in Supplementary Tables S5–S8.

**Table 2:** The number of differentially abundant proteins found for each comparison.

Treatment Comparison	Total differentially abundant proteins (No.)	More abundant proteins (No.)	Less abundant proteins (No.)
EV vs NT	28	8	20
WT vs NT	52	11	41
MUT vs NT	61	31	30
MUT vs WT	37	16	21

EV: empty vector transfected cells; NT: non-transfected cells; MUT: mutant transfected cells; WT: wild-type transfected cells.

Each protein set was uploaded to both KEGG^8^ (Kanehisa et al., 2021) and STRING^9^ (Szklarczyk et al., 2019) for pathway analysis. KEGG examines which pathways the proteins in each set are involved in, whereas STRING identifies the pathways that both the proteins and their immediate interactors are involved in.

**Figure 2:**
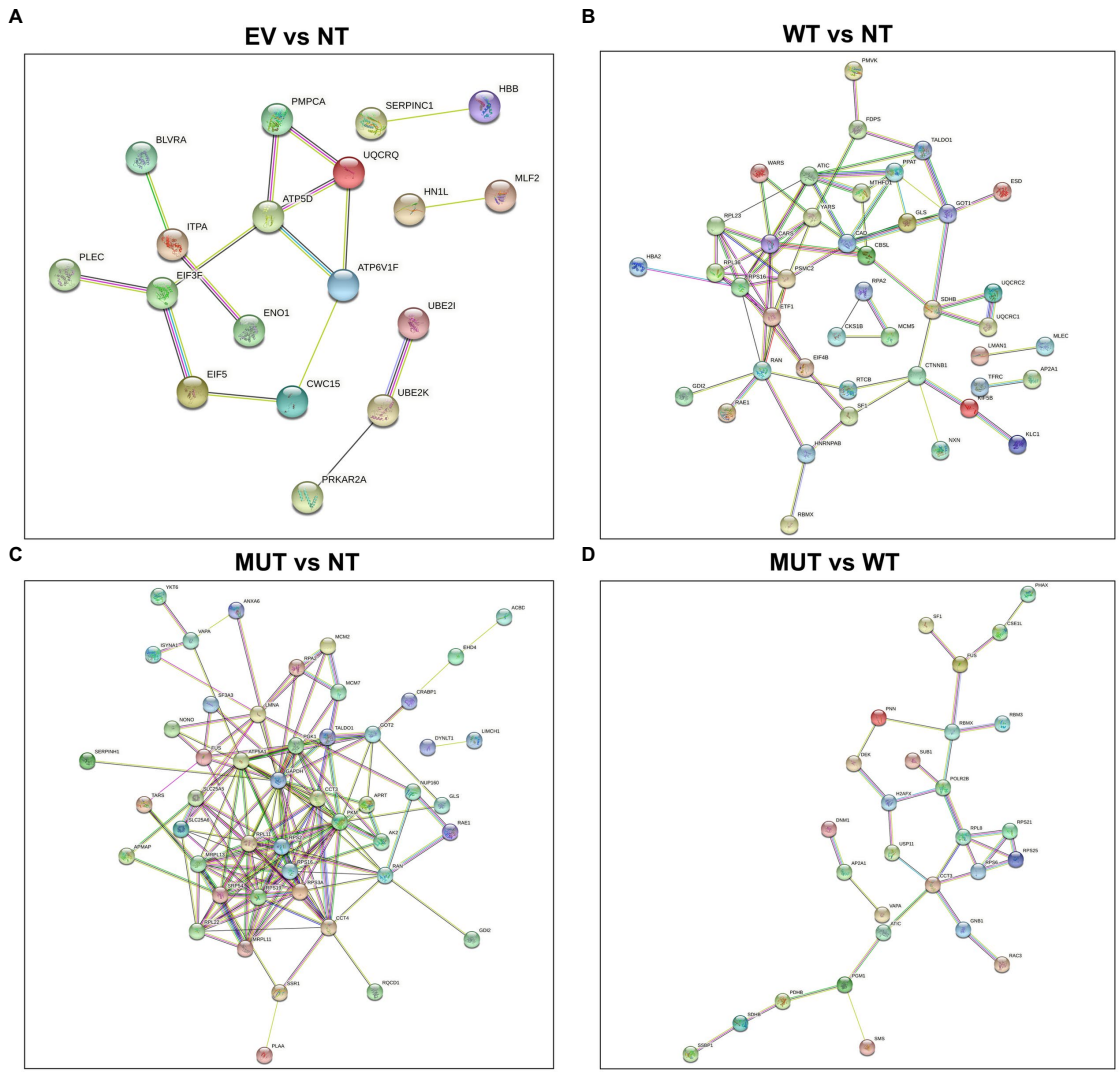
STRING protein–protein interaction diagrams of the differentially abundant proteins. **(A)** EV vs NT, **(B)** WT vs NT, **(C)** MUT vs NT, **(D)** MUT vs WT. Proteins which do not form part of an interaction network have been excluded. STRING: https://string-db.org (Szklarczyk et al., 2019). EV: empty vector transfected cells, MUT: mutant transfected cells; NT: non-transfected cells; WT: wild-type transfected cells.

STRING interaction diagrams are shown in Figure 2. As can be seen in the EV vs NT analysis (Figure 2A), there are not many interactions and most consist of only a few nodes. This shows that these protein networks are likely enriched by chance. The MUT vs NT analysis (Figure 2C) has the largest number of interactions, showing the potential of the MUT to influence cellular pathways. In the main analysis (MUT vs WT; Figure 2D), multiple proteins are shown to interact in succession, hinting that there is a single mode of action for the MUT NRXN2α. Each analysis using STRING also provided a set of enrichment terms which show which molecular mechanisms the generated protein network is involved in.

Enrichment terms from STRING were combined with those from KEGG and are shown in Table 3. “Metabolic pathways” are enriched across all analyses, suggesting that any treatment could have an effect on the general cellular metabolic pathways. In the EV vs NT analysis, “Alzheimer disease,” “Huntington disease,” and “Parkinson disease” were enriched. However, the same three proteins (QCR8, ATPD, and SNCA) were present in each group, showing that this may be a chance finding. “Alzheimer disease,” “Amyotrophic lateral sclerosis,” “Huntington disease,” “prion disease,” and “Parkinson disease” were enriched in both the WT vs NT and MUT vs NT analyses. When examining the MUT vs WT, the only enrichment term related to neurodegenerative disorders was “Huntington disease.” However, “ribosome,” “spliceosome,” “mRNA surveillance pathway,” and “nucleocytoplasmic transport” were also enriched in the MUT vs WT analysis. This may hint toward a mode of action of the mutant protein whereby protein translation and transport are affected by its overexpression.

**Table 3:** Enrichment terms obtained from the KEGG and STRING online tools for each protein set.

EV vs NT (no. of proteins)	WT vs NT (no. of proteins)	MUT vs NT (no. of proteins)	MUT vs WT (no. of proteins)
Metabolic pathways (7)	Metabolic pathways (15)	Metabolic pathways (12)	Metabolic pathways (5)
Alzheimer’s disease (4)	Alzheimer’s disease (7)	Ribosome (8)	Coronavirus disease—COVID-19 (4)
Pathways of neurodegeneration—multiple diseases (3)	Amyotrophic lateral sclerosis (7)	Amyotrophic lateral sclerosis (5)	Ribosome (4)
Huntington’s disease (3)	Huntington’s disease (7)	Alzheimer’s disease (5)	Alcoholism (3)
Oxidative phosphorylation (3)	Parkinson’s disease (6)	Biosynthesis of amino acids (5)	Diabetic cardiomyopathy (3)
Parkinson’s disease (3)	Prion disease (6)	Carbon metabolism (5)	Huntington’s disease (3)
	Alanine, aspartate and glutamate metabolism (4)	Pathways of neurodegeneration— multiple diseases (5)	mRNA Surveillance Pathway (3)
	Aminoacyl-tRNA biosynthesis (3)	Salmonella infection (5)	Nucleocytoplasmic transport (3)
	Cysteine and methionine metabolism (3)	Diabetic cardiomyopathy (4)	Spliceosome (3)
	Carbon metabolism (3)	DNA replication (4)	
		Huntington’s disease (4)	
		Parkinson’s disease (4)	
		Prion disease (4)	

KEGG: https://www.kegg.jp (Kanehisa et al., 2021), STRING: https://string-db.org (Szklarczyk et al., 2019). EV: empty vector transfected cells; NT: non-transfected cells, MUT: mutant transfected cells; and WT: wild-type transfected cells.

In the MUT vs WT analysis, the only enrichment term related to neurodegenerative disorders was “Huntington disease.” The three proteins involved in this pathway were AP2A1, RPB2, and SDHB. STRING analysis shows that these proteins are not predicted to interact with each other and do not form part of the same networks. Therefore, this enrichment is more likely to be a random result of introducing cDNA to the cells. However, “ribosome,” “spliceosome,” “mRNA surveillance pathway,” and “nucleocytoplasmic transport” were also enriched in this analysis. RPL8, RPS6, RPS21, and RPS25 are shown to be part of the “ribosome.” XPO2, PHAX, and PNN are involved in “nucleocytoplasmic transport.” FUS, PNN, and PP1B are involved in the “mRNA surveillance pathway.” FUS, RBMX, and PRP4 are involved in the “spliceosome.” RPL8, RPS6, RPS21, and RPS25 are all ribosomal subunits, involved in ribosomal and mRNA pathways.

STRING analysis shows that PHAX, XPO2, FUS, RBMX, and PNN potentially interact in sequence. PHAX is a phosphoprotein adaptor involved in RNA export and XPO2 plays a role in protein import/export within the nucleus. FUS and RBMX are both RNA-binding proteins. FUS plays a role in processes such as transcription regulation, RNA splicing, RNA transport, DNA repair, and damage responses, while RBMX plays several roles in the regulation of pre- and post-transcriptional processes. PNN is a transcriptional activator for the E-cadherin promoter gene, but it is also involved in RNA binding and mRNA splicing via the spliceosome. PRP4 is a U4/U6 small nuclear ribonucleoprotein which participates in pre-mRNA splicing. PP1B is a serine/threonine-protein phosphatase. Protein phosphatases are essential for cell division, and PP1B participates in the regulation of glycogen metabolism, muscle contractility, and protein synthesis. It has also been shown to be involved in the mRNA surveillance pathway. Taken together, the pathways of these proteins all relate back to the regulation of transcription, strongly suggesting that this process is being affected in the MUT cells.

### Enrichment analysis

WebGestalt facilitates the uploading of a gene or protein set and the corresponding fold changes and then provides enriched Gene Ontology (GO) terms. The graphical results of this analysis are shown in Supplementary Figure S3. “Negative enrichment” scores refer to GO terms that are downregulated or suppressed, while “positive enrichment” scores refer to GO terms that are upregulated or promoted. No specific enrichment was observed for the EV vs NT analysis, suggesting that transfecting the cells with the empty vector had a minimal effect.

In the WT vs NT analysis, the highest positive enrichment was for “aminoacyl-tRNA biosynthesis.” Interestingly, “oxidative phosphorylation,” “Alzheimer disease” and “Parkinson disease” also showed positive enrichment scores. The mitochondrial proteins QCR1, QCR2, and SDHB were the ones enriched in all these pathways, again showing that the mitochondrial dysfunction may be affected in the WT cells.

In the MUT vs NT analysis, “metabolic pathways” was positively enriched with the increased abundance of NADC, a protein involved in the catabolism of quinolinic acid. In the MUT vs WT analysis “Huntington disease” and “metabolic pathways” were negatively enriched and had false discovery rates (FDRs) lower than 0.05. RPB2, SPSY, SDHB, PGM1, PUR9, and ODPB were negatively enriched for “metabolic pathways.” All these proteins, except RPB2, are involved in carboxylic acid metabolism. AP2A1 and RPB2 were negatively enriched in “Huntington disease.” AP2A1 is part of the adaptor protein complex 2 which functions in protein transport via transport vesicles. It is also involved in endolysosomal trafficking and is thus implicated in several neurodegenerative disorders (Müller, 2014; Heaton et al., 2020; Srinivasan et al., 2022). RPB2 is a subunit of DNA-dependent RNA polymerase II, and therefore, its main function is in RNA transcription.

Findings from our study suggest that transfecting cells with the plasmids is having an effect on ribosomal processes. Therefore, enrichment scores for the GO term “ribosome” across each analysis have been summarized in Supplementary Figure S4. In the WT vs NT analysis, only this GO term had an FDR ≤ 0.05, showing that it highly likely that it has been negatively enriched in this protein set. In the MUT vs NT analysis, “ribosome” was again negatively enriched and had an FDR ≤ 0.05. However, in the MUT vs WT analysis, “ribosome” was positively enriched, but its FDR was above 0.05. Still, dysregulated ribosomal functioning seems to be common among the analyses and may be an important biological process related to the NRXN2α protein.

## Discussion

This exploratory analysis has revealed that wild-type NRXN2α may play a role in pathways related to neurodegenerative disorders. Since the transfection efficiency and NRXN2α levels between the WT and MUT were similar, we can be relatively confident that the proteomics analysis showed differences caused by the overexpressed proteins and not by other technical differences between the two groups. In addition, while overexpression of the empty vector plasmid did show similar enrichment terms to the other analyses (Table 2), when performing enrichment analysis for the EV transfected cells vs NT cells, it can be seen that none of these terms were significantly enriched (Supplementary Figure S3A). Therefore, we postulate that the EV only had a minimal effect on the cells and the majority of changes in the other analyses are in fact due to the NRXN2α cDNA insert (WT or MUT).

Overexpression of the WT protein in SH-SY5Y cells led to the enrichment of proteins involved in neurodegenerative diseases, such as Alzheimer’s disease, Amyotrophic lateral sclerosis, and Parkinson’s disease. In particular, the enriched proteins were involved in mitochondrial and lysosomal functioning, which are known to be dysregulated in PD and other neurodegenerative disorders (Rego and Oliveira, 2003; Wang et al., 2018). Thus, the wild-type protein may be involved in pathways related to the development of neurodegenerative disorders, such as PD. This provides further evidence potentially implicating synaptic proteins in the pathobiology of PD. Overexpression of the MUT NRXN2α-CFP protein showed similar results. Proteins unique to the MUT cells were enriched for terms related to “ribosome.” In addition, when directly comparing the WT transfected cells with the MUT transfected cells, terms related to “ribosome” were enriched. This may thus hint at a mode of action for the p.G849D mutant protein. Since the main function of the ribosome is translation of mRNA into protein, dysregulated translation could be implicated as a biological process involved in neurodegeneration. Furthermore, both cytoplasmic and mitochondrial ribosomal proteins were enriched. Indeed, it has been shown that if synaptic translation is dysregulated, mitochondrial physiology can be altered (Kuzniewska et al., 2020). In addition, EIF4G1, another protein implicated in PD, is known to be involved in protein translation processes (Chartier-Harlin et al., 2011). Furthermore, the DJ-1 and SYNJ1 proteins implicated in PD (Bonifati et al., 2003; Krebs et al., 2013) also have RNA-binding functions. DJ-1 acts to protect cells from oxidative stress and cell death by acting as an oxidative stress sensor and redox-sensitive chaperone and protease, while SYNJ1 is a phosphatase involved in synaptic vesicle endocytosis and neurotransmitter transport. A few studies have additionally identified mitochondrial ribosomal proteins in PD. Gaare et al. (2018) identified *MRPL4*, which encodes a component of the large mitochondrial ribosome subunit, in an analysis of two PD cohorts, while Billingsley et al. (2019) identified *MRPL43* and *MRPS34*, encoding components of the large and small mitochondrial ribosome subunits, using data from a PD genome-wide association study (GWAS). Both these studies thus link mtDNA translation to PD risk. Dysregulated mRNA translation can therefore be considered to play a role in PD pathogenesis (Martin, 2016). In addition, a recent RNA-sequencing analysis showed that there was differential expression of ribosomal-related pathways in their PD cohort (Hemmings et al., 2022). Therefore, it is plausible that synaptic translation could also be important in PD pathogenesis. Here, changes in translation could affect oxidative stress and the transport of neurotransmitters, thereby causing cells to be more susceptible to cell damage and death. In addition, a study on lymphoblasts generated from PD patients showed an overall downregulation of genes involved in protein synthesis (Annesley et al., 2022). Thus, recent literature has shown that dysregulated synaptic translation and mitochondrial dysfunction are linked (Kuzniewska et al., 2020), mitochondrial ribosomal proteins have been linked in a PD GWAS (Billingsley et al., 2019), pathways related to ribosomes are enriched in an RNA-sequencing analysis of a PD cohort (Hemmings et al., 2022), and that lymphoblasts generated from PD patients have dysregulated expression of genes involved in protein synthesis (Annesley et al., 2022). In addition, some of the known PD-associated proteins are also shown to have RNA or protein translation roles. This link between mRNA translation is poorly understood, but a few reviews have highlighted that restoring translation and proteostasis might be a useful target for new therapeutics (Correddu and Leung, 2019; Zhou et al., 2019). Impaired proteostasis at the synapse could also be important for PD (Nachman and Verstreken, 2022) while reduced synaptic activity and dysregulated extracellular matrix pathways have recently been reported in midbrain neurons from PD patients, providing evidence that synaptopathy is a general phenotype in PD (Stern et al., 2022). Thus, biological processes related to the ribosome, translation, and tRNA, specifically at the synapse, could possibly be an important molecular mechanism in PD pathobiology.

The strength of this study is that we examined the effect of overexpression of both the wild-type and mutant protein using a hypothesis-free approach. In this way, we were able to show that potential mode of action of the mutant protein but were also able to conclude that the wild-type protein is also involved in pathways related to neurodegeneration. Therefore, it is possible that any dysregulation of NRXN2α could potentially lead to neurodegeneration.

However, we also acknowledge several limitations, including the use of a commercial cell line for this study. While SH-SY5Y cells are good *in vitro* model for PD as they display a catecholaminergic phenotype, producing both dopamine and noradrenaline (Xicoy et al., 2017), there are always limitations when using cell lines to study a complex human disorder. Unfortunately, we were not able to obtain dermal fibroblast samples from the individuals harboring the *NRXN2* variant as an *ex-vivo* model for this study. We also acknowledge the limitations of overexpressing a murine gene in a human cell line. Therefore, in the future it would be important to repeat these experiments in fibroblasts from the patients or in animal models. Another limitation is the use of shotgun proteomics. Since this study is explorative, we investigated the total proteome to determine which biological pathways were being affected. However, it may be important to do more targeted proteomics work in future, such as looking into post-translational modifications as well as investigating phospho-proteomics to determine signaling changes. Indeed, several kinases and phosphatases were observed in the different analyses, therefore phospho-proteomics would be required to better understand the effect of these protein changes.

In conclusion, findings from this exploratory study possibly implicate the NRXN2α protein in neurodegenerative processes and show that synaptic ribosomal and translation processes may be important in PD and/ or other neurodegenerative disorders. However, further validation of NRXN2α and the proteins implicated in synaptic ribosomal and translation processes in other models of PD or neurodegenerative disorders would be required to prove or disprove this hypothesis.

## Data availability statement

The mass spectrometry proteomics data have been deposited to the ProteomeXchange Consortium via the PRIDE partner repository with the dataset identifier PXD036636 and 10.6019/ PXD036636.

## Ethics statement

Ethical approval was obtained from the Health Research Ethics Committee (Protocol numbers 2002/C059 and S20/01/005 PhD) and the Research Ethics Committee: Biological and Environmental Safety (Protocol number BEE-2021-13149). Both committees are located at Stellenbosch University, Cape Town, South Africa.

## Author contributions

KC conducted all experiments, performed all analyses, and wrote the first draft of the manuscript. SF assisted with analysis of the mass spectrometry data. AM-N assisted with data processing. MV performed the protein extraction and mass spectrometry. RC assisted with writing and editing of the manuscript. KC and SB conceptualized the study and acquired funding. All authors contributed to the article and approved the submitted version.

## Funding

This work is based on the research supported in part by the National Research Foundation of South Africa (NRF) (Grant Numbers: 129249 and 146254); the South African Medical Research Council (SAMRC) (self-initiated research grant); the Harry Crossley Foundation and Stellenbosch University, South Africa. SAMRC and The Higher Education Department, Next Generation of Academic Programme (nGAP), provided support for RC in the form of a fulltime academic position and salary.

## Conflict of interest

The authors declare that the research was conducted in the absence of any commercial or financial relationships that could be construed as a potential conflict of interest.

## Publisher’s note

All claims expressed in this article are solely those of the authors and do not necessarily represent those of their affiliated organizations, or those of the publisher, the editors and the reviewers. Any product that may be evaluated in this article, or claim that may be made by its manufacturer, is not guaranteed or endorsed by the publisher.

## Footnotes

^1^ https://www.thegpm.org/crap

^2^ https://bioinfogp.cnb.csic.es/tools/venny

^3^ https://www.uniprot.org

^4^ https://www.kegg.jp

^5^ https://string-db.org

^6^ http://www.webgestalt.org

^7^ https://string-db.org

^8^ https://www.kegg.jp

^9^ https://string-db.org

## References

[ref1] Annesley S. J. Allan C. Y. Sanislav O. Evans A. Fisher P. R. (2022). Dysregulated gene expression in Lymphoblasts from Parkinson’s disease. Proteomes 10:20. doi: 10.3390/proteomes10020020, PMID: 35736800 PMC9230639

[ref2] Billingsley K. J. Barbosa I. A. Bandrés-Ciga S. Quinn J. P. Bubb V. J. Deshpande C.. (2019). Mitochondria function associated genes contribute to Parkinson’s disease risk and later age at onset. NPJ Parkinsons Dis. 5, 1–9. doi: 10.1038/s41531-019-0080-x, PMID: 31123700 PMC6531455

[ref3] Bonifati V. Rizzu P. Van Baren M. J. Schaap O. Breedveld G. J. Krieger E.. (2003). Mutations in the DJ-1 gene associated with autosomal recessive early-onset Parkinsonism. Science 299, 256–259. doi: 10.1126/science.1077209 12446870

[ref4] Chartier-Harlin M. C. Dachsel J. C. Vilariño-Güell C. Lincoln S. J. Leprêtre F. Hulihan M. M.. (2011). Translation initiator EIF4G1 mutations in familial Parkinson disease. Am. J. Hum. Genet. 89, 398–406. doi: 10.1016/j.ajhg.2011.08.009, PMID: 21907011 PMC3169825

[ref5] Correddu D. Leung I. K. H. (2019). Targeting mRNA translation in Parkinson’s disease. Drug Discov. Today 24, 1295–1303. doi: 10.1016/j.drudis.2019.04.003 30974176

[ref6] Craig A. M. Kang Y. (2007). Neurexin-neuroligin signaling in synapse development. Curr. Opin. Neurobiol. 17, 43–52. doi: 10.1016/j.conb.2007.01.011, PMID: 17275284 PMC2820508

[ref7] Dachtler J. Ivorra J. L. Rowland T. E. Lever C. John Rodgers R. Clapcote S. J. (2015). Heterozygous deletion of α-neurexin I or α-neurexin II results in behaviors relevant to autism and schizophrenia. Behav. Neurosci. 129, 765–776. doi: 10.1037/bne0000108, PMID: 26595880 PMC4655861

[ref8] Dorsey E. R. Elbaz A. Nichols E. Abd-Allah F. Abdelalim A. Adsuar J. C.. (2018). Global, regional, and national burden of Parkinson’s disease, 1990–2016: a systematic analysis for the global burden of disease study 2016. Lancet Neurol. 17, 939–953. doi: 10.1016/S1474-4422(18)30295-3, PMID: 30287051 PMC6191528

[ref9] Feigin V. L. Krishnamurthi R. V. Theadom A. M. Abajobir A. A. Mishra S. R. Ahmed M. B.. (2017). Global, regional, and national burden of neurological disorders during 1990–2015: a systematic analysis for the global burden of disease study 2015. Lancet Neurol. 16, 877–897. doi: 10.1016/S1474-4422(17)30299-5, PMID: 28931491 PMC5641502

[ref10] Gaare J. J. Nido G. S. Sztromwasser P. Knappskog P. M. Dahl O. Lund-Johansen M.. (2018). Rare genetic variation in mitochondrial pathways influences the risk for Parkinson’s disease. Mov. Disord. 33, 1591–1600. doi: 10.1002/mds.64, PMID: 30256453 PMC6282592

[ref11] Heaton G. R. Landeck N. Mamais A. Nalls M. A. Nixon-Abell J. Kumaran R.. (2020). Sequential screening nominates the Parkinson’s disease associated kinase LRRK2 as a regulator of Clathrin-mediated endocytosis. Neurobiol. Dis. 141:104948. doi: 10.1016/j.nbd.2020.104948, PMID: 32434048 PMC7339134

[ref12] Hemmings S. M. J. Swart P. Womersely J. S. Ovenden E. S. van den Heuvel L. L. McGregor N. W.. (2022). RNA-seq analysis of gene expression profiles in posttraumatic stress disorder, Parkinson’s disease and schizophrenia identifies roles for common and distinct biological pathways. Discov. Ment. Heal. 2, 1–18. doi: 10.1007/s44192-022-00009-y PMC10501040 37861850

[ref13] Kanehisa M. Furumichi M. Sato Y. Ishiguro-Watanabe M. Tanabe M. (2021). KEGG: integrating viruses and cellular organisms. Nucleic Acids Res. 49, D545–D551. doi: 10.1093/nar/gkaa970, PMID: 33125081 PMC7779016

[ref14] Kang Y. Zhang X. Dobie F. Wu H. Craig A. M. (2008). Induction of GABAergic postsynaptic differentiation by α-neurexins. J. Biol. Chem. 283, 2323–2334. doi: 10.1074/jbc.M703957200, PMID: 18006501 PMC2811689

[ref15] Krebs C. E. Karkheiran S. Powell J. C. Cao M. Makarov V. Darvish H.. (2013). The sac1 domain of SYNJ1 identified mutated in a family with early-onset progressive Parkinsonism with generalized seizures. Hum. Mutat. 34, 1200–1207. doi: 10.1002/humu.22372, PMID: 23804563 PMC3790461

[ref16] Kuzniewska B. Cysewski D. Wasilewski M. Sakowska P. Milek J. Kulinski T. M.. (2020). Mitochondrial protein biogenesis in the synapse is supported by local translation. EMBO Rep. 21:e48882. doi: 10.15252/embr.201948882, PMID: 32558077 PMC7403725

[ref17] Liao Y. Wang J. Jaehnig E. J. Shi Z. Zhang B. (2019). WebGestalt 2019: gene set analysis toolkit with revamped UIs and APIs. Nucleic Acids Res. 47, W199–W205. doi: 10.1093/nar/gkz401, PMID: 31114916 PMC6602449

[ref18] Martin I. (2016). Decoding Parkinson’s disease pathogenesis: the role of deregulated mRNA translation. J. Parkinsons Dis. 6, 17–27. doi: 10.3233/JPD-150738, PMID: 26889638 PMC4927901

[ref19] Missler M. Zhang W. Rohlmann A. Kattenstroth G. Hammer R. E. Gottmann K.. (2003). α-neurexins couple Ca 2+ channels to synaptic vesicle exocytosis. Nature 423, 939–948. doi: 10.1038/nature01755, PMID: 12827191

[ref20] Müller S. (2014). In silico analysis of regulatory networks underlines the role of miR-10b-5p and its target BDNF in huntington’s disease. Transl. Neurodegener. 3, 1–5. doi: 10.1186/2047-9158-3-17, PMID: 25210621 PMC4160136

[ref21] Nachman E. Verstreken P. (2022). Synaptic proteostasis in Parkinson’s disease. Curr. Opin. Neurobiol. 72, 72–79. doi: 10.1016/j.conb.2021.09.001, PMID: 34653835

[ref22] Oliveros J. C. (2015). Venny. An interactive tool for comparing lists with Venn’s diagrams. Available at: https://bioinfogp.cnb.csic.es/tools/venny/

[ref23] Panicker N. Ge P. Dawson V. L. Dawson T. M. (2021). The cell biology of Parkinson’s disease. J. Cell Biol. 220:e202012095. doi: 10.1083/jcb.202012095, PMID: 33749710 PMC8103423

[ref24] Rego A. C. Oliveira C. R. (2003). Mitochondrial dysfunction and reactive oxygen species in excitotoxicity and apoptosis: implications for the pathogenesis of neurodegenerative diseases. Neurochem. Res. 28, 1563–1574. doi: 10.1023/A:1025682611389 14570402

[ref25] Searle B. C. (2010). Scaffold: a bioinformatic tool for validating MS/MS-based proteomic studies. Proteomics 10, 1265–1269. doi: 10.1002/pmic.200900437, PMID: 20077414

[ref26] Sebate B. Cuttler K. Cloete R. Britz M. Christoffels A. Williams M.. (2021). Prioritization of candidate genes for a south African family with Parkinson’s disease using in-silico tools. PLoS One 16:e0249324. doi: 10.1371/journal.pone.0249324, PMID: 33770142 PMC7997022

[ref27] Srinivasan S. Gal J. Bachstetter A. Nelson P. T. (2022). Alpha adaptins show isoform-specific association with neurofibrillary tangles in Alzheimer’s disease. Neuropathol. Appl. Neurobiol. 48:e12776. doi: 10.1111/nan.12776, PMID: 34820873 PMC8810620

[ref28] Stern S. Lau S. Manole A. Rosh I. Percia M. Ezer R. B.. (2022). Reduced synaptic activity and dysregulated extracellular matrix pathways are common phenotypes in midbrain neurons derived from sporadic and mutation-associated Parkinson’s disease patients. NPJ Parkinsons Dis. 8:103. doi: 10.1038/S41531-022-00366-Z, PMID: 35948563 PMC9365794

[ref29] Südhof T. C. (2008). Neuroligins and neurexins link synaptic function to cognitive disease. Nature 455, 903–911. doi: 10.1038/nature07456, PMID: 18923512 PMC2673233

[ref30] Szklarczyk D. Gable A. L. Lyon D. Junge A. Wyder S. Huerta-Cepas J.. (2019). STRING v11: protein-protein association networks with increased coverage, supporting functional discovery in genome-wide experimental datasets. Nucleic Acids Res. 47, D607–D613. doi: 10.1093/nar/gky1131, PMID: 30476243 PMC6323986

[ref31] The UniProt Consortium (2021). UniProt: the universal protein knowledgebase in 2021. Nucleic Acids Res. 49, D480–D489. doi: 10.1093/nar/gkaa1100, PMID: 33237286 PMC7778908

[ref32] Wang C. Telpoukhovskaia M. A. Bahr B. A. Chen X. Gan L. (2018). Endo-lysosomal dysfunction: a converging mechanism in neurodegenerative diseases. Curr. Opin. Neurobiol. 48, 52–58. doi: 10.1016/j.conb.2017.09.005, PMID: 29028540

[ref33] Xicoy H. Wieringa B. Martens G. J. M. (2017). The SH-SY5Y cell line in Parkinson’s disease research: a systematic review. Mol. Neurodegener. 12, 10–11. doi: 10.1186/s13024-017-0149-0, PMID: 28118852 PMC5259880

[ref34] Yakhine-Diop S. Martínez-Chacón G. Uribe-Carretero E. Niso-Santano M. González-Polo R. Fuentes J. (2019). The paradigm of protein acetylation in Parkinson’s disease. Neural Regen. Res. 14, 975–976. doi: 10.4103/1673-5374.250575, PMID: 30762005 PMC6404488

[ref35] Zhou Z. D. Selvaratnam T. Lee J. C. T. Chao Y. X. Tan E. K. (2019). Molecular targets for modulating the protein translation vital to proteostasis and neuron degeneration in Parkinson’s disease. Transl. Neurodegener. 8, 6–14. doi: 10.1186/s40035-019-0145-0, PMID: 30740222 PMC6360798

