# Supplementary material for: Proteomics analysis of the p.G849D variant in neurexin 2 alpha may reveal insight into Parkinson’s disease pathobiology
Source: Front Aging Neurosci. 2022 Nov 30;14:1002777. doi: 10.3389/fnagi.2022.1002777 (PMC9748613; doi:10.3389/fnagi.2022.1002777)
Supplement: Supplementary file 1 [file Data_Sheet_1.docx]

Supplementary Figures


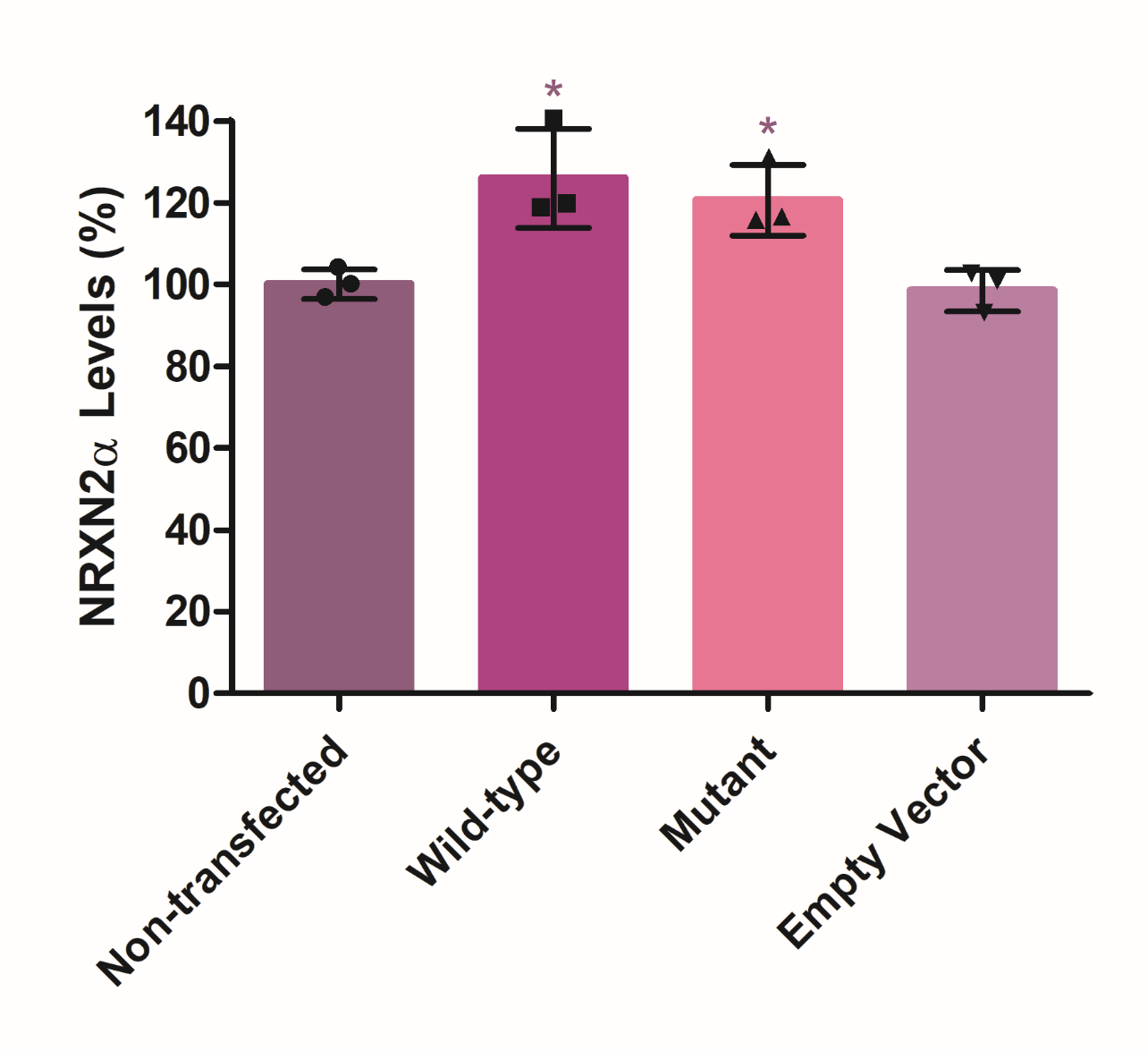


**Figure S1: Overexpression of wild-type and mutant NRXN2α in SH-SY5Y cells increases NRXN2α protein levels.** Flow cytometric analysis of NRXN2α levels shows an increase in NRXN2α levels in both the wild-type (p = 0.03) and mutant (p = 0.02) transfected cells when compared to the non-transfected cells. There is no significant change in the empty vector (p = 0.70) transfected cells compared to non-transfected cells. *n* = 3; one-way ANOVA and student’s *t*-test.

**A)**

**B)**

**C)**

**D)**

**Supplementary Figure S2: Total Ion Chromatograms (TICs) indicate successful digestion of peptides.**

a) EV, b) NT, c) WT, d) MUT. Individual TICs refer to the sample triplicates. The x-axis represents Time and the y-axis represents the Relative Abundance of the peptides. The same amount of sample was injected and each TIC shows similar intensities in the range 4 E9-7E9. Abbreviations: EV: empty vector transfected cells; FDR: false discovery rate; MUT: mutant transfected cells; NT: non-transfected cells; WT: wild-type transfected cells.

**A)**


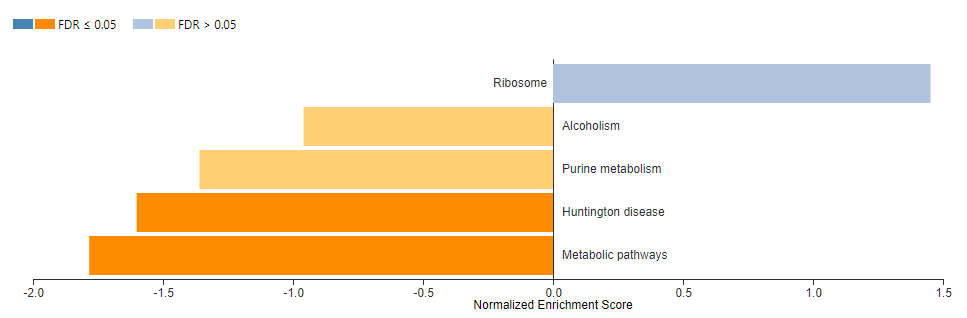

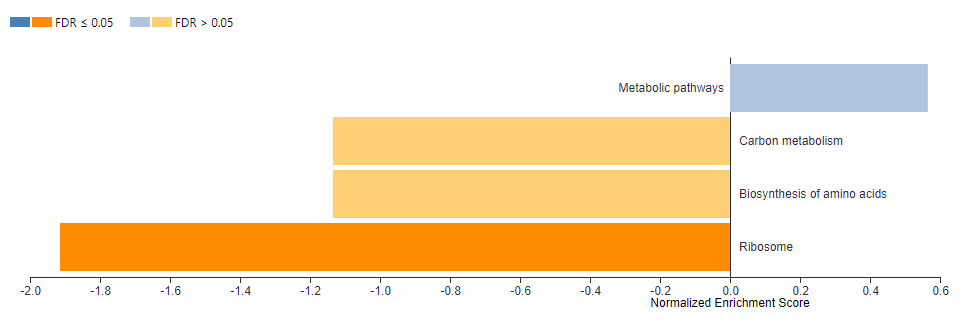

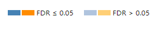

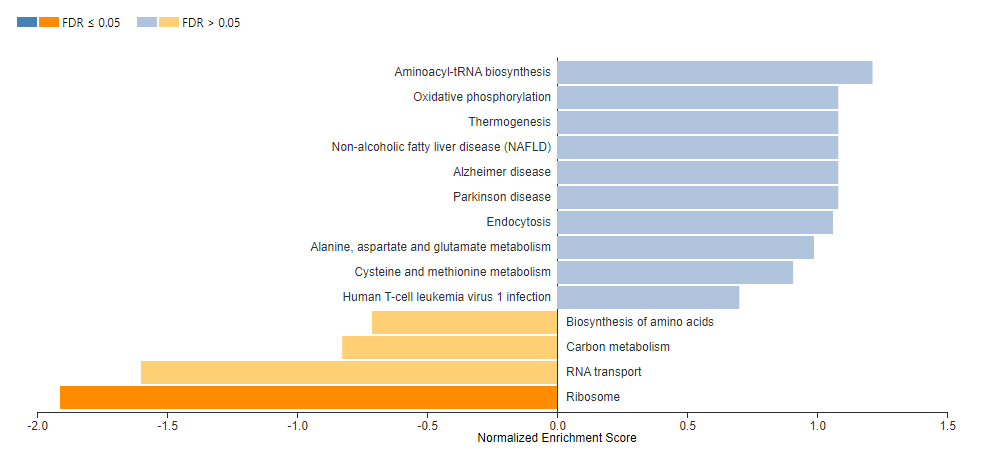


**D)**

**C)**

**B)**


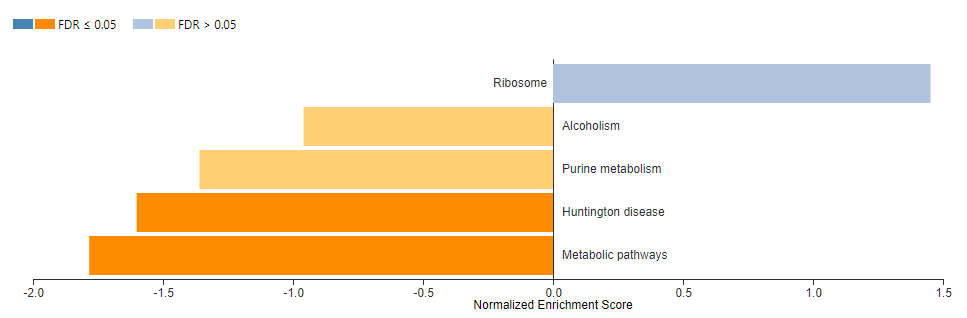

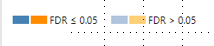


**Figure S3: Gene set enrichment analysis performed using WebGestalt (**[**http://www.webgestalt.org**](http://www.webgestalt.org)**).**

EV vs NT (a), WT vs NT (b), MUT vs NT (c), and MUT vs WT (d). Gene Ontology terms in orange are negatively enriched, while those in blue are positively enriched. Abbreviations: EV: empty vector transfected cells; FDR: false discovery rate; MUT: mutant transfected cells; NT: non-transfected cells; WT: wild-type transfected cells. WebGestalt: Liao *et al.*, 2019


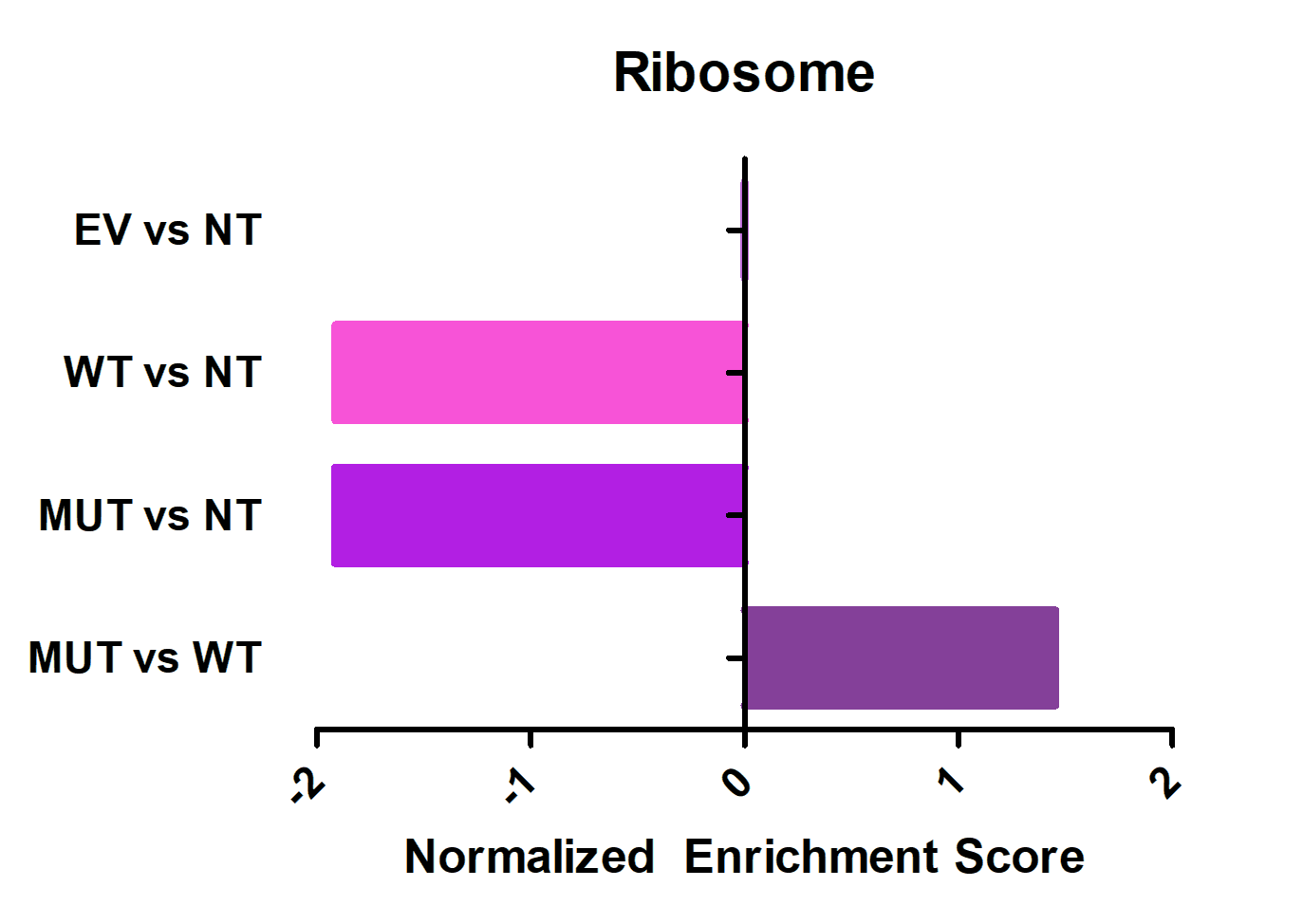
**Figure S4: Normalized expression scores obtained from WebGestalt (**[**http://www.webgestalt.org**](http://www.webgestalt.org)**) for the GO term “ribosome” in each analysis.**

Abbreviations: EV: empty vector transfected cells; NT: non-transfected cells; MUT: mutant transfected cells; WT: wild-type transfected cells. WebGestalt: Liao *et al.*, 2019
