# Supplementary material for: Proteomics analysis of the p.G849D variant in neurexin 2 alpha may reveal insight into Parkinson’s disease pathobiology
Source: Front Aging Neurosci. 2022 Nov 30;14:1002777. doi: 10.3389/fnagi.2022.1002777 (PMC9748613; doi:10.3389/fnagi.2022.1002777)
Supplement: Supplementary file 2 [file Data_Sheet_2.docx]

Supplementary Methods

# Plasmids

## Site-directed Mutagenesis

# In order to generate the p.G849D mutant plasmid (p.G882D in our mouse model (mouse genomic position: 540693_chr19 (GRCm39)), site-directed mutagenesis was performed on the wild-type NRXN2α-ECFP-N1 plasmid using the Q5 Site-Directed Mutagenesis kit (New England Biolabs) as per the manufacturer’s instructions. Briefly, the wild-type plasmid was used as template DNA in a PCR reaction with a mutagenic forward primer, containing the single base pair change that leads to the generation of the G to D substitution, and back-to-back reverse primer. The primer sequences are as follows: Q5 G882D Forward: *5’-GTG TTC AAT GAT CAA CCC TAC ATG GAC C-3’*; Q5 G882D Reverse: *5’-CAG CCC ACT CAG GTG CCC-3’*. The PCR reaction consisted of an initial denaturation at 98 °C for 30 seconds, followed by 25 cycles of 98 °C for 10 seconds, 69 °C for 30 seconds and 72 °C for 5 minutes, and a final extension at 72 °C for 2 minutes. Following the PCR reaction, the kinase, ligase and DpnI enzyme mixture was added to the PCR product and incubated at room temperature for five minutes. This mixture is used to efficiently phosphorylate, ligate, and circularize the new construct and remove the template construct. After the incubation period, 5 μl of the reaction mixture was used for bacterial transformation of the NEB 5-alpha competent *E. coli* cells. Confirmation of mutagenesis was performed using Sanger sequencing of plasmid DNA at Stellenbosch University’s Central Analytical Facilities (CAF).

## Plasmid DNA Isolation

After successful transformation of competent *E. coli*, colonies were inoculated in a 5 ml starter culture of LB media supplemented with 5 μl kanamycin sulfate (50 mg/ml). The starter culture was incubated at 37 °C for 8 hours in a shaking incubator set at 200 rpm. Thereafter, 100 μl of the starter culture was added to 100 ml of LB media supplemented with 100 μl kanamycin sulfate (50 mg/ml). This culture was incubated at 37 °C for 16 hours in a shaking incubator set at 200 rpm. The culture was then centrifuged at 3 400 x *g* to pellet the cells. Plasmid DNA was isolated from the bacteria using the ZymoPURE II Plasmid MidiPrep Kit (Zymo Research Corp.) as per the manufacturer’s centrifugation protocol. Isolated plasmid DNA was stored at -20 °C until required.

# Transfection

SH-SY5Y cells were grown in sterile 25 cm^3^ flasks until 70% confluent and transfected using Lipofectamine3000 (Invitrogen) as per the manufacturer’s instructions Briefly, 5 μg plasmid DNA was added to 10 μl P3000 in 250 μl serum free media (SFM). 7.5 μl Lipofectamine3000 was prepared separately in 250 μl SFM. Thereafter, the two volumes were combined, gently mixed, and incubated for 10-20 minutes at room temperature. DMEM supplemented with 15% FBS without penicillin/streptomycin was then added to each well. The combined transfection reagents were then added dropwise to each flask and the flasks were incubated at 37 °C, 5% CO_2_. After 24 hours, the media was removed and replaced with pre-warmed complete media.

# NRXN2α Levels

Prior to proteomics analysis, NRXN2α protein levels were determined using immunofluorescent flow cytometry to confirm overexpression of NRXN2α. Briefly, cells were seeded at a density of 500 000 cells per well in a 6-well plate and transfected with the wild-type, mutant, or empty vector plasmids. After 24 hours the media was removed, and cells were trypsinized and transferred to 1.5 ml Eppendorf tubes. Cell pellets were harvested by centrifugation at 438 x *g* for 10 minutes. The cells were fixed using 3% formaldehyde in PBS for 15 minutes and permeabilized using 0.25% Triton X-100 in PBS for 15 minutes. Cells were then blocked with 0.5% BSA in PBS for 10 minutes and resuspended in 1:100 rabbit anti-NRXN2 primary antibody (Abcam: ab34245) in PBS. These suspensions were incubated overnight at 4 °C. The following morning, the cells were washed in PBS by centrifuging at 2739 x *g* for 5 minutes and resuspended in 1:100 anti-rabbit Cy3 secondary antibody (Jackson ImmunoResearch Laboratories: 111-165-003) in PBS. These suspensions were incubated in the dark at room temperature for 2 hours. Cells were washed twice in PBS by centrifuging at 2739 x *g* for 5 minutes and resuspended in deionized water for analysis with the Guava® Muse® Cell Analyzer (Luminex). Unstained, non-transfected cells and stained, non-transfected cells were used to set the gating parameters. Readings were obtained using the Open Module Red option with 5000 events recorded for each sample. Median fluorescence values were used for analysis of NRXN2α levels.

# Proteomics Analysis

## Sample Cleanup

Extraction reagents were removed using a chloroform-methanol-water liquid-liquid extraction method. The samples (100 μl) were mixed with 400 μl methanol and thoroughly mixed before the addition of 100 μl chloroform. Again, the samples were thoroughly mixed before the addition of 400 μl type I water. After mixing the samples were centrifuged for 3 minutes at 13 000 x *g* to induce phase separation. The top phase was removed and 400 μl methanol was added and the sample mixed again. The samples were centrifuged again for 3 minutes at 13 000 x *g* to pellet the protein wafer that formed during the phase partition.

For the on bead digest, samples were re-suspended in 50 mM NH_4_HCO_3_ before reduction with 5 mM triscarboxyethyl phosphine (TCEP; Fluka) in 100 mM NH_4_HCO_3_ for 1 hour at room temperature with agitation. Cysteine residues were thiomethylated with 20 mM *S*-Methyl methanethiosulfonate (Sigma) in 50 mM NH_4_HCO_3_ for 30 minutes at room temperature. After thiomethylation the samples were diluted two-fold with binding buffer (100 mM Ammonium acetate, 30% acetonitrile, pH 4.5). The protein solution was added to MagResyn HILIC magnetic particles prepared according to manufacturer’s instructions and incubated for 3 hours with rotation. After binding the supernatant was removed and the magnetic particles washed twice with washing buffer (100 mM Ammonium acetate, 95% acetonitrile, pH 4.5). After washing the magnetic particles were suspended in 10 mM NH_4_HCO_3_ containing trypsin (Pierce) to a final ratio of 1:50. After an overnight incubation at 37 °C the peptides were extracted once with 50 μl water 1% trifluoroacetic acid (TFA). The samples were dried down and re-suspended in 30 μl 2% acetonitrile:water; 0.1% formic acid (FA).

## Liquid Chromatography

Liquid chromatography was performed on a Thermo Scientific Ultimate 3000 RSLC equipped with a 20 mm x 100 μm C_18_ trap column (Thermo Fisher) and a CSH 25 cm x 75 μm, 1.7μm particle size C_18_ column (Waters) analytical column. The solvent system employed was loading: 2% acetonitrile:water; 0.1% FA; Solvent A: 2% acetonitrile:water; 0.1% FA and Solvent B: 100% acetonitrile:water. The samples were loaded onto the trap column using loading solvent at a flow rate of 2 μl/min from a temperature controlled autosampler set at 7 C. Loading was performed for 5 minutes before the sample was eluted onto the analytical column. Flow rate was set to 300 nl/min and the gradient generated as follows: 2% from 0-5 minutes, 2% - 30% from 5 to 65 minutes and 30 - 50% B from 65-80 minutes. Chromatography was performed at 45 °C and the outflow delivered to the mass spectrometer.
