# Supplementary material for: Proteomics analysis of the p.G849D variant in neurexin 2 alpha may reveal insight into Parkinson’s disease pathobiology
Source: Front Aging Neurosci. 2022 Nov 30;14:1002777. doi: 10.3389/fnagi.2022.1002777 (PMC9748613; doi:10.3389/fnagi.2022.1002777)
Supplement: Supplementary file 3 [file Data_Sheet_3.docx]

Supplementary Tables

**Supplementary Table S1: List of unique proteins in wild-type transfected cells**

| **Protein** | **Name** | **Function** |
| --- | --- | --- |
| sp\|P0DTE8\|AMY1C_HUMAN | Alpha-amylase 1C | Calcium-binding enzyme that initiates starch digestion in the oral cavity. |
| sp\|Q9HD20\|AT131_HUMAN | Endoplasmic reticulum transmembrane helix translocase | Endoplasmic reticulum translocase required to remove mitochondrial transmembrane proteins mistargeted to the endoplasmic reticulum. |
| sp\|O60885\|BRD4_HUMAN | Bromodomain-containing protein 4 | Chromatin reader protein that recognizes and binds acetylated histones and plays a key role in transmission of epigenetic memory across cell divisions and transcription regulation. |
| sp\|Q9Y315\|DEOC_HUMAN | Deoxyribose-phosphate aldolase | Catalyzes a reversible aldol reaction between acetaldehyde and D-glyceraldehyde 3-phosphate to generate 2-deoxy-D-ribose 5-phosphate. Participates in stress granule (SG) assembly. May allow ATP production from extracellular deoxyinosine in conditions of energy deprivation. |
| sp\|O60610\|DIAP1_HUMAN | Protein diaphanous homolog 1 | Actin nucleation and elongation factor required for the assembly of F-actin structures, such as actin cables and stress fibers. |
| sp\|Q9H4M9\|EHD1_HUMAN | EH domain-containing protein 1 | ATP- and membrane-binding protein that controls membrane reorganization/tubulation upon ATP hydrolysis. |
| sp\|Q96A65\|EXOC4_HUMAN | Exocyst complex component 4 | Component of the exocyst complex involved in the docking of exocytic vesicles with fusion sites on the plasma membrane. |
| sp\|Q9UJY5\|GGA1_HUMAN | ADP-ribosylation factor-binding protein GGA1 | Plays a role in protein sorting and trafficking between the trans-Golgi network (TGN) and endosomes. |
| sp\|P04899\|GNAI2_HUMAN | Guanine nucleotide-binding protein G | Guanine nucleotide-binding proteins (G proteins) are involved as modulators or transducers in various transmembrane signaling systems. |
| sp\|Q8NBJ5\|GT251_HUMAN | Procollagen galactosyltransferase 1 | Beta-galactosyltransferase that transfers beta-galactose to hydroxylysine residues of type I collagen. |
| sp\|Q9BZE4\|GTPB4_HUMAN | GTP-binding protein 4 | Involved in the biogenesis of the 60S ribosomal subunit. |
| sp\|Q12907\|LMAN2_HUMAN | Vesicular integral-membrane protein VIP36 | Plays a role as an intracellular lectin in the early secretory pathway. |
| sp\|Q5VWZ2\|LYPL1_HUMAN | Lysophospholipase-like protein | Has depalmitoylating activity toward KCNMA1. Able to hydrolyze only short chain substrates due to its shallow active site. |
| sp\|Q86U44\|MTA70_HUMAN | N6-adenosine-methyltransferase catalytic subunit | The METTL3-METTL14 heterodimer forms a N6-methyltransferase complex that methylates adenosine residues at the N(6) position of some RNAs and regulates various processes such as the circadian clock, differentiation of embryonic and hematopoietic stem cells, cortical neurogenesis, response to DNA damage, differentiation of T-cells and primary miRNA processing. |
| sp\|O60287\|NPA1P_HUMAN | Nucleolar pre-ribosomal-associated protein 1 | Involved in RNA binding. |
| sp\|Q8WVJ2\|NUDC2_HUMAN | NudC domain-containing protein 2 | May regulate the LIS1/dynein pathway by stabilizing LIS1 with Hsp90 chaperone. |
| sp\|O60313\|OPA1_HUMAN | Dynamin-like 120 kDa protein, mitochondrial | Dynamin-related GTPase that is essential for normal mitochondrial morphology by regulating the equilibrium between mitochondrial fusion and mitochondrial fission. |
| sp\|O43913\|ORC5_HUMAN | Origin recognition complex subunit 5 | Component of the origin recognition complex (ORC) that binds origins of replication. |
| sp\|O15428\|PINL_HUMAN | Putative PIN1-like protein | Enzyme with peptidyl-prolyl cis-trans isomerase activity. |
| sp\|Q9UKA9\|PTBP2_HUMAN | Polypyrimidine tract-binding protein 2 | RNA-binding protein which binds to intronic polypyrimidine tracts and mediates negative regulation of exons splicing. |
| sp\|Q02127\|PYRD_HUMAN | Dihydroorotate dehydrogenase | Catalyzes the conversion of dihydroorotate to orotate with quinone as electron acceptor. |
| sp\|P20742\|PZP_HUMAN | Pregnancy zone protein | Is able to inhibit all four classes of proteinases by a unique 'trapping' mechanism. |

**Supplementary Table S2: List of unique proteins in mutant transfected cells**

| **Protein** | **Name** | **Function** |
| --- | --- | --- |
| sp\|P05067\|A4_HUMAN | Amyloid-beta precursor protein | Functions as a cell surface receptor and performs physiological functions on the surface of neurons relevant to neurite growth, neuronal adhesion and axonogenesis. Interaction between APP molecules on neighboring cells promotes synaptogenesis. |
| sp\|O94805\|ACL6B_HUMAN | Actin-like protein 6B | Involved in transcriptional activation and repression of select genes by chromatin remodeling (alteration of DNA-nucleosome topology). |
| sp\|O60678\|ANM3_HUMAN | Protein arginine N-methyltransferase 3 | Protein-arginine N-methyltransferase that catalyzes both the monomethylation and asymmetric dimethylation of the guanidino nitrogens of arginine residues in target proteins, and therefore falls into the group of type I methyltransferase. May regulate retinoic acid synthesis and signaling by inhibiting ALDH1A1 retinal dehydrogenase activity. |
| sp\|Q13873\|BMPR2_HUMAN | Bone morphogenetic protein receptor type-2 | On ligand binding, forms a receptor complex consisting of two type II and two type I transmembrane serine/threonine kinases. Type II receptors phosphorylate and activate type I receptors which autophosphorylate, then bind and activate SMAD transcriptional regulators. |
| sp\|Q15059\|BRD3_HUMAN | Bromodomain-containing protein 3 | Chromatin reader that recognizes and binds hyperacetylated chromatin and plays a role in the regulation of transcription, probably by chromatin remodeling and interaction with transcription factors. Regulates transcription by promoting the binding of the transcription factor GATA1 to its targets. |
| sp\|Q9HC52\|CBX8_HUMAN | Chromobox protein homolog 8 | Component of a Polycomb group (PcG) multiprotein PRC1-like complex, a complex class required to maintain the transcriptionally repressive state of many genes, including Hox genes, throughout development. PcG PRC1 complex acts via chromatin remodeling and modification of histones; it mediates monoubiquitination of histone H2A 'Lys-119', rendering chromatin heritably changed in its expressibility. |
| sp\|Q96CT7\|CC124_HUMAN | Coiled-coil domain-containing protein 124 | Required for proper progression of late cytokinetic stages. |
| sp\|O76031\|CLPX_HUMAN | ATP-dependent Clp protease ATP-binding subunit clpX-like, mitochondrial | ATP-dependent specificity component of the Clp protease complex. Targets specific substrates for degradation by the Clp complex. |
| sp\|Q5JTJ3\|COA6_HUMAN | Cytochrome c oxidase assembly facto 6 homolog | Involved in the maturation of the mitochondrial respiratory chain complex IV subunit MT-CO2/COX2. Thereby, may regulate early steps of complex IV assembly. |
| sp\|P67870\|CSK2B_HUMAN | Casein kinase II subunit beta | Regulatory subunit of casein kinase II/CK2. As part of the kinase complex regulates the basal catalytic activity of the alpha subunit a constitutively active serine/threonine-protein kinase that phosphorylates a large number of substrates containing acidic residues C-terminal to the phosphorylated serine or threonine. |
| sp\|Q13616\|CUL1_HUMAN | Cullin-1 | Core component of multiple cullin-RING-based SCF (SKP1-CUL1-F-box protein) E3 ubiquitin-protein ligase complexes, which mediate the ubiquitination of proteins involved in cell cycle progression, signal transduction and transcription. |
| sp\|O60231_DHX16_HUMAN | Pre-mRNA-spicing factor ATP-dependent RNA helicase DHX16 | Required for pre-mRNA splicing as component of the spliceosome. |
| sp\|P09884\|DPOLA_HUMAN | DNA polymerase alpha catalytic subunit | Catalytic subunit of the DNA polymerase alpha complex (also known as the alpha DNA polymerase-primase complex) which plays an essential role in the initiation of DNA synthesis. |
| sp\|Q5T1H1\|EYS_HUMAN | Protein eyes shut homology | Required to maintain the integrity of photoreceptor cells. |
| sp\|Q8WXD5\|GEMI6_HUMAN | Gem-associated protein 6 | The SMN complex catalyzes the assembly of small nuclear ribonucleoproteins (snRNPs), the building blocks of the spliceosome, and thereby plays an important role in the splicing of cellular pre-mRNAs. |
| sp\|Q9H3K2\|GHITM_HUMAN | Growth hormone-inducible transmembrane protein | Required for the mitochondrial tubular network and cristae organization. Involved in apoptotic release of cytochrome c. |
| sp\|P34931\|HS71L_HUMAN | Heat shock 70 kDa protein 1-like | Molecular chaperone implicated in a wide variety of cellular processes, including protection of the proteome from stress, folding and transport of newly synthesized polypeptides, activation of proteolysis of misfolded proteins and the formation and dissociation of protein complexes. Plays a pivotal role in the protein quality control system, ensuring the correct folding of proteins, the re-folding of misfolded proteins and controlling the targeting of proteins for subsequent degradation. |
| sp\|Q9NX55\|HYPK_HUMAN | Huntingtin-interacting protein K | Component of several N-terminal acetyltransferase complexes. |
| sp\|Q9HA64\|KT3K_HUMAN | Ketosamine-3-kinase | Ketosamine-3-kinase involved in protein deglycation by mediating phosphorylation of ribuloselysine and psicoselysine on glycated proteins, to generate ribuloselysine-3 phosphate and psicoselysine-3 phosphate, respectively. Ribuloselysine-3 phosphate and psicoselysine-3 phosphate adducts are unstable and decompose under physiological conditions. |
| sp\|Q9H9P8\|L2HDH_HUMAN | L-2-hydroxyglutarate dehydrogenase, mitochondrial | Mitochondrial enzyme with (S)-2-hydroxy-acid oxidase activity and 2-hydroxyglutarate dehydrogenase activity. |
| sp\|Q9NX58\|LYAR_HUMAN | Cell growth-regulating nucleolar protein | Plays a role in the maintenance of the appropriate processing of 47S/45S pre-rRNA to 32S/30S pre-rRNAs and their subsequent processing to produce 18S and 28S rRNAs. Also acts at the level of transcription regulation. |
| sp\|Q9H0A0\|NAT10_HUMAN | RNA cytidine acetyltransferase | RNA cytidine acetyltransferase that catalyzes the formation of N(4)-acetylcytidine (ac4C) modification on mRNAs, 18S rRNA and tRNAs. Catalyzes ac4C modification of a broad range of mRNAs, enhancing mRNA stability and translation. |
| sp\|O00712\|NFIB_HUMAN | Nuclear factor 1 B-type | Transcriptional activator of GFAP, essential for proper brain development. |
| sp\|Q9BVI4\|NOC4L_HUMAN | Nucleolar complex protein 4 homology | Involved in RNA binding. |
| sp\|Q96P11\|NSUN5_HUMAN | 28s rRNA (cytosine-C(5))-methyltransferase | S-adenosyl-L-methionine-dependent methyltransferase that specifically methylates the C(5) position of cytosine 3782 (m5C3782) in 28S rRNA. m5C3782 promotes protein translation without affecting ribosome biogenesis and fidelity. |
| sp\|P13674\|P4HA1_HUMAN | Prolyl 4-hydroxylase subunit alpha-1 | Catalyzes the post-translational formation of 4-hydroxyproline in -Xaa-Pro-Gly- sequences in collagens and other proteins. |
| sp\|P51003\|PAPOA_HUMAN | Poly(A) polymerase alpha | Polymerase that creates the 3'-poly(A) tail of mRNAs. Also required for the endoribonucleolytic cleavage reaction at some polyadenylation sites. |
| sp\|Q9NTI5\|PDS5B_HUMAN | Sister chromatid cohesion protein PDS5 homolog B | Regulator of sister chromatid cohesion in mitosis which may stabilize cohesin complex association with chromatin. May couple sister chromatid cohesion during mitosis to DNA replication. Cohesion ensures that chromosome partitioning is accurate in both meiotic and mitotic cells and plays an important role in DNA repair. |
| sp\|Q63HM9\|PLCX3_HUMAN | PI-PLC X domain-containing protein 3 | Enzyme with phosphoric diester hydrolase activity. |
| sp\|Q86TP1\|PRUN1_HUMAN | Exopolyphosphastase PRUNE1 | Phosphodiesterase (PDE) that has higher activity toward cAMP than cGMP, as substrate. Plays a role in cell proliferation, migration and differentiation, and acts as a negative regulator of NME1. |
| sp\|Q15269\|PWP2_HUMAN | Periodic tryptophan protein 2 homology | Involved in RNA binding. |
| sp\|P20338\|RAB4A_HUMAN | Ras-related protein Rab-4A | Small GTPase which cycles between an active GTP-bound and an inactive GDP-bound state. Involved in protein transport and in vesicular traffic. |
| sp\|Q9H5N1\|RABE2_HUMAN | Rab GTPase-binding effector protein 2 | Plays a role in membrane trafficking and in homotypic early endosome fusion. |
| sp\|P35251\|RFC1_HUMAN | Replication factor C subunit 1 | The elongation of primed DNA templates by DNA polymerase delta and epsilon requires the action of the accessory proteins PCNA and activator 1. This subunit binds to the primer-template junction. |
| sp\|P19388\|RPAB1_HUMAN | DNA-directed RNA polymerases I, II, and III subunit RPABC1 | DNA-dependent RNA polymerase catalyzes the transcription of DNA into RNA using the four ribonucleoside triphosphates as substrates. Common component of RNA polymerases I, II and III which synthesize ribosomal RNA precursors, mRNA precursors and many functional non-coding RNAs, and small RNAs, such as 5S rRNA and tRNAs, respectively. |
| sp\|Q9Y399}RT02_HUMAN | 28S ribosomal protein S2, mitochondrial | Required for mitoribosome formation and stability, and mitochondrial translation. |
| sp\|Q9UBV2\|SE1L1_HUMAN | Protein sel-1 homolog 1 | Plays a role in the endoplasmic reticulum quality control (ERQC) system also called ER-associated degradation (ERAD) involved in ubiquitin-dependent degradation of misfolded endoplasmic reticulum proteins. |
| sp\|Q8WVK2\|SNR27_HUMAN | U4/U6. U5 small nuclear ribonucleoprotein 27 kDa protein | May play a role in mRNA splicing. |
| sp\|P08240\|SRPRA_HUMAN | Signal recognition particle receptor subunit alpha | Component of the signal recognition particle (SRP) complex receptor (SR). Ensures, in conjunction with the SRP complex, the correct targeting of the nascent secretory proteins to the endoplasmic reticulum membrane system. |
| sp\|Q13033\|STRN3_HUMAN | Striatin-3 | Binds calmodulin in a calcium dependent manner. May function as scaffolding or signaling protein. |
| sp\|O60220\|TIM8A_HUMAN | Mitochondrial import inner membrane translocase subunit Tim8 A | Mitochondrial intermembrane chaperone that participates in the import and insertion of some multi-pass transmembrane proteins into the mitochondrial inner membrane. |
| sp\|Q56UQ5\|TPT1L_HUMAN | TPT1-like protein | Involved in calcium ion binding. |
| sp\|O75152\|ZC11A_HUMAN | Zinc finger CCCH domain-containing protein 11A | RNA-binding protein that interacts with purine-rich sequences and is involved in nuclear mRNA export; probably mediated by association with the TREX complex. |
| sp\|Q96ME7\|ZN512_HUMAN | Zinc finger protein 512 | May be involved in transcriptional regulation. |

**Supplementary Table S3: List of unique proteins in non-transfected cells**

| **Protein** | **Name** | **Function** |
| --- | --- | --- |
| sp\|Q9ULX6\|AKP8L_HUMAN | A-kinase anchor protein 8-like | Could play a role in constitutive transport element (CTE)-mediated gene expression by association with DHX9. |
| sp\|Q75179\|ANR17_HUMAN | Ankyrin repeat domain-containing protein 2 | Could play pivotal roles in cell cycle and DNA regulation. |
| sp\|Q6PL18\|ATAD2_HUMAN | ATPase family AAA domain-containing protein 2 | May be a transcriptional coactivator of the nuclear receptor ESR1 required to induce the expression of a subset of estradiol target genes, such as CCND1, MYC and E2F1. |
| sp\|P55957\|BID_HUMAN | BH3-interacting domain death agonist | Induces caspases and apoptosis. |
| sp\|Q8WUQ7\|CATIN_HUMAN | Cactin | Involved in the regulation of innate immune response. Acts as negative regulator of Toll-like receptor, interferon-regulatory factor (IRF) and canonical NF-kappa-B signaling pathways. |
| sp\|P09669\|COX6C_HUMAN | Cytochrome c oxidase subunit 6C | Component of the cytochrome c oxidase, the last enzyme in the mitochondrial electron transport chain which drives oxidative phosphorylation. |
| sp\|Q9Y4B6\|DCAF1_HUMAN | DDB1- and CUL4-associated factor 1 | Acts both as a substrate recognition component of E3 ubiquitin-protein ligase complexes and as an atypical serine/threonine-protein kinase, playing key roles in various processes such as cell cycle, telomerase regulation and histone modification. |
| sp\|Q16698\|DECR_HUMAN | 2,4-dienoyl-CoA reductase [(3E)-enoyl-CoA-producing], mitochondrial | Auxiliary enzyme of beta-oxidation. It participates in the metabolism of unsaturated fatty enoyl-CoA esters having double bonds in both even- and odd-numbered positions in mitochondria. |
| sp\|Q01780\|EXOSX_HUMAN | Exosome component 10 | Putative catalytic component of the RNA exosome complex which has 3'->5' exoribonuclease activity and participates in a multitude of cellular RNA processing and degradation events. |
| sp\|P23142\|FBLN1_HUMAN | Fibulin-1 | Incorporated into fibronectin-containing matrix fibers. May play a role in cell adhesion and migration along protein fibers within the extracellular matrix (ECM). |
| sp\|Q8TAE8\|G45IP_HUMAN | Growth arrest and DNA damage-inducible proteins-interacting protein 1 | Acts as a negative regulator of G1 to S cell cycle phase progression by inhibiting cyclin-dependent kinases. |
| sp\|Q2TB90\|HKDC1_HUMAN | Hexokinase HKDC1 | Catalyzes the phosphorylation of hexose to hexose 6-phosphate, although at very low level compared to other hexokinases. Involved in glucose homeostasis and hepatic lipid accumulation. |
| sp\|Q9BW19\|KIFC1_HUMAN | Kinesin-like protein KIFC1 | Minus end-directed microtubule-dependent motor required for bipolar spindle formation. |
| sp\|P05771\|KPCB_HUMAN | Protein kinase C beta type | Calcium-activated, phospholipid- and diacylglycerol (DAG)-dependent serine/threonine-protein kinase involved in various cellular processes such as regulation of the B-cell receptor (BCR) signalosome, oxidative stress-induced apoptosis, androgen receptor-dependent transcription regulation, insulin signaling and endothelial cells proliferation. |
| sp\|Q9Y4Y9\|LSM5_HUMAN | U6 snRNA-associated Sm-like protein LSm5 | Plays role in pre-mRNA splicing as component of the U4/U6-U5 tri-snRNP complex that is involved in spliceosome assembly, and as component of the precatalytic spliceosome (spliceosome B complex). |
| sp\|Q86UE4\|LYRIC_HUMAN | Protein LYRIC | Down-regulates SLC1A2/EAAT2 promoter activity when expressed ectopically. Activates the nuclear factor kappa-B (NF-kappa-B) transcription factor. |
| sp\|Q96S90\|LYSM1_HUMAN | LysM and putative peptidoglycan-binding domain-containing protein 1 | Enables protein binding. |
| sp\|Q9NQX4\|MYO5C_HUMAN | Unconventional myosin-Vc | May be involved in transferrin trafficking. Likely to power actin-based membrane trafficking in many physiologically crucial tissues. |
| sp\|Q9H1E3\|NUCKS_HUMAN | Nuclear ubiquitous casein and cyclin-dependent kinase substrate 1 | Chromatin-associated protein involved in DNA repair by promoting homologous recombination (HR). |
| sp\|P50479\|PDLI4_HUMAN | PDZ and LIM domain protein 4 | Suppresses SRC activation by recognizing and binding to active SRC and facilitating PTPN13-mediated dephosphorylation of SRC 'Tyr-419' leading to its inactivation. Inactivated SRC dissociates from this protein allowing the initiation of a new SRC inactivation cycle. Involved in reorganization of the actin cytoskeleton. |
| sp\|O00541\|PESC_HUMAN | Pescadillo homolog | Component of the PeBoW complex, which is required for maturation of 28S and 5.8S ribosomal RNAs and formation of the 60S ribosome. |
| sp\|P50336\|PPOX_HUMAN | Protoporphyrinogen oxidase | Catalyzes the 6-electron oxidation of protoporphyrinogen-IX to form protoporphyrin-IX. |
| sp\|P48634\|PRC2A_HUMAN | Protein PRRC2A | May play a role in the regulation of pre-mRNA splicing. |
| sp\|Q15397\|PUM3_HUMAN | Pumilio homolog 3 | Inhibits the poly(ADP-ribosyl)ation activity of PARP1 and the degradation of PARP1 by CASP3 following genotoxic stress. |
| sp\|Q6DKI1\|RL7L_HUMAN | 60S ribosomal protein L7-like 1 | Structural constituent of ribosome, involved in RNA binding. |
| sp\|Q13084\|RM28_HUMAN | 39S ribosomal protein L28, mitochondrial | Structural constituent of ribosome, involved in RNA binding. |
| sp\|P62841\|RS15_HUMAN | 40S ribosomal protein S15 | Structural constituent of ribosome, involved in RNA binding, DNA binding, MDM2/MDM4 family protein binding, ubiquitin ligase inhibitor activity. |
| sp\|Q9NP81\|SYSM_HUMAN | Serine—tRNA ligase, mitochondrial | Catalyzes the attachment of serine to tRNA(Ser). |
| sp\|O15164\|TIF1A_HUMAN | Transcription intermediary factor 1-alpha | Transcriptional coactivator that interacts with numerous nuclear receptors and coactivators and modulates the transcription of target genes. |
| sp\|Q08AM6\|VAC14_HUMAN | Protein VAC14 homolog | Scaffold protein component of the PI(3,5)P2 regulatory complex which regulates both the synthesis and turnover of phosphatidylinositol 3,5-bisphosphate (PtdIns(3,5)P2). |
| sp\|QWIWA0\|WDR75_HUMAN | WD repeat-containing protein 75 | Ribosome biogenesis factor. Involved in nucleolar processing of pre-18S ribosomal RNA. Required for optimal pre-ribosomal RNA transcription by RNA polymerase I. |

**Supplementary Table S4: List of unique proteins in empty vector transfected cells**

| **Protein** | **Name** | **Function** |
| --- | --- | --- |
| sp\|Q96IU4\|ABHEB_HUMAN | Protein ABHD14B | Has hydrolase activity towards p-nitrophenyl butyrate (in vitro). May activate transcription. |
| sp\|Q6ZN18\|AEBP2_HUMAN | Zinc finger protein AEBP2 | Acts as an accessory subunit for the core Polycomb repressive complex 2 (PRC2), which mediates histone H3K27 (H3K27me3) trimethylation on chromatin leading to transcriptional repression of the affected target gene. |
| sp\|P43652\|AFAM_HUMAN | Afamin | Functions as carrier for hydrophobic molecules in body fluids. Essential for the solubility and activity of lipidated Wnt family members. |
| sp\|Q8TD16\|BICD2_HUMAN | Protein bicaudal D homolog 2 | Acts as an adapter protein linking the dynein motor complex to various cargos and converts dynein from a non-processive to a highly processive motor in the presence of dynactin. |
| sp\|Q8TDN6\|BRX1_HUMAN | Ribosome biogenesis protein BRX1 homolog | Required for biogenesis of the 60S ribosomal subunit. |
| sp\|Q08554\|DSC1_HUMAN | Desmocollin-1 | Component of intercellular desmosome junctions. Involved in the interaction of plaque proteins and intermediate filaments mediating cell-cell adhesion. |
| sp\|O75477\|ERLN1_HUMAN | Erlin-1 | Component of the ERLIN1/ERLIN2 complex which mediates the endoplasmic reticulum-associated degradation (ERAD) of inositol 1,4,5-trisphosphate receptors (IP3Rs). |
| sp\|Q5T3I0\|GPTC4_HUMAN | G patch domain-containing protein 4 | Involved in the regulation of cell growth and nucleolar structure. |
| sp\|P07203\|GPX1_HUMAN | Glutathione peroxidase 1 | Protects the hemoglobin in erythrocytes from oxidative breakdown. |
| sp\|P02008\|HBAZ_HUMAN | Hemoglobin subunit zeta | The zeta chain is an alpha-type chain of mammalian embryonic hemoglobin. |
| sp\|P02042\|HBD_HUMAN | Hemoglobin subunit delta | Involved in oxygen transport from the lung to the various peripheral tissues. |
| sp\|Q9Y3E1\|HDGR3_HUMAN | Hepatoma-derived growth factor-related protein 3 | Enhances DNA synthesis and may play a role in cell proliferation. |
| sp\|Q9NP66\|HM20A_HUMAN | High mobility group protein 20A | Plays a role in neuronal differentiation as chromatin-associated protein. |
| sp\|P19013\|K2C4_HUMAN | Keratin, type II cytoskeletal 4 | Involved in cytoskeleton organization, epithelial cell differentiation and negative regulation of epithelial cell proliferation. |
| sp\|Q8N1N4\|K2C78_HUMAN | Keratin, type II cytoskeletal 78 | Protein with an intermediate filament domain. Keratins are the major structural proteins in epithelial cells. |
| sp\|Q13554\|KCC2B_HUMAN | Calcium/calmodulin-dependent protein kinase type II subunit beta | Calcium/calmodulin-dependent protein kinase that functions autonomously after Ca(2+)/calmodulin-binding and autophosphorylation, and is involved in dendritic spine and synapse formation, neuronal plasticity and regulation of sarcoplasmic reticulum Ca(2+) transport in skeletal muscle. |
| sp\|Q16626\|MEA1_HUMAN | Male-enhanced antigen 1 | May play an important role in spermatogenesis and/or testis development. |
| sp\|Q9UHG2\|PCS1N_HUMAN | ProSAAS | May function in the control of the neuroendocrine secretory pathway. |
| sp\|A2A3N6\|PIPSL_HUMAN | Putative PIP5K1A and PSMD4-like protein | Has negligible PIP5 kinase activity. Binds to ubiquitinated proteins. |
| sp\|P35813\|PPM1A_HUMAN | Protein phosphatase 1A | Enzyme with a broad specificity. Negatively regulates TGF-beta signaling through dephosphorylating SMAD2 and SMAD3, resulting in their dissociation from SMAD4, nuclear export of the SMADs and termination of the TGF-beta-mediated signaling. |
| sp\|P06702\|S10A9_HUMAN | Protein S100-A9 | S100A9 is a calcium- and zinc-binding protein which plays a prominent role in the regulation of inflammatory processes and immune response. |
| sp\|Q5PRF9\|SMAG2_HUMAN | Protein Smaug homolog 2 | Has transcriptional repressor activity. Overexpression inhibits the transcriptional activities of AP-1, p53/TP53 and CDKN1A. |
| sp\|Q9H4B7\|TBB1_HUMAN | Tubulin beta-1 chain | Tubulin is the major constituent of microtubules. |
| sp\|Q5QJE6\|TDIF2_HUMAN | Deoxynucleotidyltransferase terminal-interacting protein 2 | Regulates the transcriptional activity of DNTT and ESR1. May function as a chromatin remodeling protein. |
| sp\|Q96EK4\|THA11_HUMAN | THAP domain-containing protein 11 | Transcriptional repressor that plays a central role for embryogenesis and the pluripotency of embryonic stem (ES) cells. |
| sp\|Q86VY4\|TSYL5_HUMAN | Testis-specific T-encoded-like protein 5 | Involved in modulation of cell growth and cellular response to gamma radiation probably via regulation of the Akt signaling pathway. |
| sp\|Q9H832\|UBE2Z_HUMAN | Ubiquitin-conjugating enzyme E2 Z | Catalyzes the covalent attachment of ubiquitin to other proteins. |
| sp\|P16989\|YBOX3_HUMAN | Y-box-binding protein 3 | Binds to the GM-CSF promoter. Seems to act as a repressor. May have a role in translation repression. |

**Supplementary Table S5: List of proteins differentially abundant between wild-type transfected and non-transfected cells**

| **Fold Change** | **Protein** | **Name** | **Function** |
| --- | --- | --- | --- |
| **Less Abundant** | | | |
| -1.8566911 | sp\|Q9Y3U8\|RL36_HUMAN | 60S ribosomal protein 36 | Component of the large ribosomal subunit. |
| -1.6788144 | sp\|Q86Y82\|STX12_HUMAN | Syntaxin-12 | SNARE that acts to regulate protein transport between late endosomes and the trans-Golgi network. The SNARE complex containing STX6, STX12, VAMP4 and VTI1A mediates vesicle fusion |
| -1.121847 | sp\|Q15637\|SF01_HUMAN | Splicing factor 1 | Necessary for the ATP-dependent first step of spliceosome assembly. Binds to the intron branch point sequence (BPS) 5'-UACUAAC-3' of the pre-mRNA. May act as transcription repressor. |
| -1.0410199 | sp\|Q9Y520\|PRC2C_HUMAN | Protein PRRC2C | Required for efficient formation of stress granules. |
| -0.74689996 | sp\|P38159\|RBMX_HUMAN | RNA-binding motif protein, X chromosome | RNA-binding protein that plays several role in the regulation of pre- and post-transcriptional processes. Implicated in tissue-specific regulation of gene transcription and alternative splicing of several pre-mRNAs. |
| -0.6849414 | sp\|P23588\|IF4B_HUMAN | Eukaryotic translation initiation factor 4B | Required for the binding of mRNA to ribosomes |
| -0.5337738 | sp\|P53999\|TCP4_HUMAN | Activated RNA polymerase II transcriptional coactivator p15 | General coactivator that functions cooperatively with TAFs and mediates functional interactions between upstream activators and the general transcriptional machinery. May be involved in stabilizing the multiprotein transcription complex. Binds single-stranded DNA. |
| -0.50896925 | sp\|P37837\|TALDO_HUMAN | Transaldolase | Transaldolase is important for the balance of metabolites in the pentose-phosphate pathway. |
| -0.37183204 | sp\|P78406\|RAE1L_HUMAN | mRNA export factor | Plays a role in mitotic bipolar spindle formation. Binds mRNA. May function in nucleocytoplasmic transport and in directly or indirectly attaching cytoplasmic mRNPs to the cytoskeleton. |
| -0.3331039 | sp\|P62826\|RAN_HUMAN | GTP-binding nuclear protein Ran | GTPase involved in nucleocytoplasmic transport, participating both to the import and the export from the nucleus of proteins and RNAs |
| -0.30670425 | sp\|Q01518\|CAP1_HUMAN | Adenylyl cyclase-associated protein 1 | Directly regulates filament dynamics and has been implicated in a number of complex developmental and morphological processes, including mRNA localization and the establishment of cell polarity. |
| **More Abundant** | | | |
| 0.33976412 | sp\|P31939\|PUR9_HUMAN | Bifunctional purine biosynthesis protein ATIC | Bifunctional enzyme that catalyzes the last two steps of purine biosynthesis |
| 0.35881603 | sp\|Q07866\|KLC1_HUMAN | Kinesin light chain 1 | Kinesin is a microtubule-associated force-producing protein that may play a role in organelle transport. The light chain may function in coupling of cargo to the heavy chain or in the modulation of its ATPase activity. |
| 0.37648672 | sp\|P11586\|C1TC_HUMAN | C-1-tetrahydrofolate synthase, cytoplasmic | This protein is involved in the pathway tetrahydrofolate interconversion, which is part of One-carbon metabolism. |
| 0.38646522 | sp\|P27708\|PYR1_HUMAN | CAD protein | This protein is a 'fusion' protein encoding four enzymatic activities of the pyrimidine pathway (GATase, CPSase, ATCase and DHOase). |
| 0.3885932 | sp\|P62495\|ERF1_HUMAN | Eukaryotic peptide chain release factor subunit 1 | Directs the termination of nascent peptide synthesis (translation) in response to the termination codons UAA, UAG and UGA. |
| 0.41403818 | sp\|P69905\|HBA_HUMAN | Hemoglobin subunit alpha | Involved in oxygen transport from the lung to the various peripheral tissues. |
| 0.42103648 | sp\|P50395\|GDIB_HUMAN | Rab GDP dissociation inhibitor beta | Regulates the GDP/GTP exchange reaction of most Rab proteins by inhibiting the dissociation of GDP from them, and the subsequent binding of GTP to them. |
| 0.4262979 | sp\|P10768\|ESTD_HUMAN | S-formylglutathione hydrolase | Serine hydrolase involved in the detoxification of formaldehyde. |
| 0.43407574 | sp\|Q9UBB4\|ATX10_HUMAN | Ataxin-10 | Necessary for the survival of cerebellar neurons. Induces neuritogenesis by activating the Ras-MAP kinase pathway. May play a role in the maintenance of a critical intracellular glycosylation level and homeostasis. |
| 0.43839556 | sp\|Q9Y3I0\|RTCB_HUMAN | RNA-splicing ligase RtcB homolog | Catalytic subunit of the tRNA-splicing ligase complex that acts by directly joining spliced tRNA halves to mature-sized tRNAs by incorporating the precursor-derived splice junction phosphate into the mature tRNA as a canonical 3',5'-phosphodiester. May act as an RNA ligase with broad substrate specificity, and may function toward other RNAs. |
| 0.50248647 | sp\|P02786\|TFR1_HUMAN | Transferrin receptor protein 1 | Cellular uptake of iron occurs via receptor-mediated endocytosis of ligand-occupied transferrin receptor into specialized endosomes. Acts as a lipid sensor that regulates mitochondrial fusion by regulating activation of the JNK pathway. |
| 0.5164809 | sp\|P35998\|PRS7_HUMAN | 26S proteasome regulatory subunit 7 | Component of the 26S proteasome, a multiprotein complex involved in the ATP-dependent degradation of ubiquitinated proteins. |
| 0.5284708 | sp\|P0DN79\|CBSL_HUMAN | Cystathionine beta-synthase-like protein | Hydro-lyase catalyzing the first step of the transsulfuration pathway, where the hydroxyl group of L-serine is displaced by L-homocysteine in a beta-replacement reaction to form L-cystathionine, the precursor of L-cysteine. This catabolic route allows the elimination of L-methionine and the toxic metabolite L-homocysteine. Also involved in the production of hydrogen sulfide, a gasotransmitter with signaling and cytoprotective effects on neurons. |
| 0.53512836 | sp\|P23381\|SYWC_HUMAN | Tryptophan--tRNA ligase, cytoplasmic | Regulates ERK, Akt, and eNOS activation pathways that are associated with angiogenesis, cytoskeletal reorganization and shear stress-responsive gene expression. |
| 0.54786944 | sp\|Q99873\|ANM1_HUMAN | Protein arginine N-methyltransferase 1 | Arginine methyltransferase that methylates (mono and asymmetric dimethylation) the guanidino nitrogens of arginyl residues present in proteins such as ESR1, histone H2, H3 and H4, ILF3, HNRNPA1, HNRNPD, NFATC2IP, SUPT5H, TAF15, EWS, HABP4 and SERBP1 |
| 0.57735336 | sp\|Q14141\|SEPT6_HUMAN | Septin-6 | Filament-forming cytoskeletal GTPase. Required for normal organization of the actin cytoskeleton. Involved in cytokinesis. |
| 0.58534056 | sp\|P14324\|FPPS_HUMAN | Farnesyl pyrophosphate synthase | Key enzyme in isoprenoid biosynthesis which catalyzes the formation of farnesyl diphosphate (FPP), a precursor for several classes of essential metabolites including sterols, dolichols, carotenoids, and ubiquinones. |
| 0.59459674 | sp\|Q9BT78\|CSN4_HUMAN | COP9 signalosome complex subunit 4 | Component of the COP9 signalosome complex (CSN), a complex involved in various cellular and developmental processes. The CSN complex is an essential regulator of the ubiquitin (Ubl) conjugation pathway. |
| 0.6055922 | sp\|P21912\|SDHB_HUMAN | Succinate dehydrogenase [ubiquinone] iron-sulfur subunit, mitochondrial | Iron-sulfur protein (IP) subunit of succinate dehydrogenase (SDH) that is involved in complex II of the mitochondrial electron transport chain and is responsible for transferring electrons from succinate to ubiquinone (coenzyme Q). |
| 0.61219233 | sp\|P17174\|AATC_HUMAN | Aspartate aminotransferase, cytoplasmic | Biosynthesis of L-glutamate from L-aspartate or L-cysteine. Important regulator of levels of glutamate, the major excitatory neurotransmitter of the vertebrate central nervous system. Acts as a scavenger of glutamate in brain neuroprotection. |
| 0.61734223 | sp\|P33992\|MCM5_HUMAN | DNA replication licensing factor MCM5 | Acts as component of the MCM2-7 complex (MCM complex) which is the putative replicative helicase essential for 'once per cell cycle' DNA replication initiation and elongation in eukaryotic cells. |
| 0.6257046 | sp\|P54577\|SYYC_HUMAN | Tyrosine--tRNA ligase, cytoplasmic | Catalyzes the attachment of tyrosine to tRNA(Tyr) in a two-step reaction: tyrosine is first activated by ATP to form Tyr-AMP and then transferred to the acceptor end of tRNA(Tyr). |
| 0.71930486 | sp\|P33176\|KINH_HUMAN | Kinesin-1 heavy chain | Microtubule-dependent motor required for normal distribution of mitochondria and lysosomes. |
| 0.72144926 | sp\|P31930\|QCR1_HUMAN | Cytochrome b-c1 complex subunit 1, mitochondrial | Component of the ubiquinol-cytochrome c oxidoreductase, a multisubunit transmembrane complex that is part of the mitochondrial electron transport chain which drives oxidative phosphorylation. |
| 0.7647878 | sp\|P49257\|LMAN1_HUMAN | Protein ERGIC-53 | Mannose-specific lectin. May recognize sugar residues of glycoproteins, glycolipids, or glycosylphosphatidyl inositol anchors and may be involved in the sorting or recycling of proteins, lipids, or both. |
| 0.8274879 | sp\|P22695\|QCR2_HUMAN | Cytochrome b-c1 complex subunit 2, mitochondrial | Component of the ubiquinol-cytochrome c oxidoreductase, a multisubunit transmembrane complex that is part of the mitochondrial electron transport chain which drives oxidative phosphorylation. |
| 0.88206655 | sp\|Q06203\|PUR1_HUMAN | Amidophosphoribosyl- transferase | This protein is involved in step **1** of the subpathway that synthesizes N(1)-(5-phospho-D-ribosyl)glycinamide from 5-phospho-alpha-D-ribose 1-diphosphate. This subpathway is part of the pathway IMP biosynthesis via de novo pathway, which is itself part of Purine metabolism. |
| 0.8948843 | sp\|P01023\|A2MG_HUMAN | Alpha-2-macroglobulin | Is able to inhibit all four classes of proteinases by a unique 'trapping' mechanism. |
| 0.913311 | sp\|P52788\|SPSY_HUMAN | Spermine synthase | Catalyzes the production of spermine from spermidine and decarboxylated S-adenosylmethionine (dcSAM). |
| 0.93997234 | sp\|Q15631\|TSN_HUMAN | Translin | DNA-binding protein that specifically recognizes consensus sequences at the breakpoint junctions in chromosomal translocations, mostly involving immunoglobulin (Ig)/T-cell receptor gene segments. Seems to recognize single-stranded DNA ends generated by staggered breaks occurring at recombination hot spots. Exhibits both single-stranded and double-stranded endoribonuclease activity. May act as an activator of RNA-induced silencing complex (RISC) by facilitating endonucleolytic cleavage of the siRNA passenger strand. |
| 0.9789583 | sp\|P35222\|CTNB1_HUMAN | Catenin beta-1 | Key downstream component of the canonical Wnt signaling pathway. |
| 1.062929 | sp\|O94905\|ERLN2_HUMAN | Erlin-2 | Component of the ERLIN1/ERLIN2 complex which mediates the endoplasmic reticulum-associated degradation (ERAD) of inositol 1,4,5-trisphosphate receptors (IP3Rs) such as ITPR1. |
| 1.1214811 | sp\|Q15126\|PMVK_HUMAN | Phosphomevalonate kinase | Catalyzes the reversible ATP-dependent phosphorylation of mevalonate 5-phosphate to produce mevalonate diphosphate and ADP, a key step in the mevalonic acid mediated biosynthesis of isopentenyl diphosphate and other polyisoprenoid metabolites. |
| 1.1282995 | sp\|O43172\|PRP4_HUMAN | U4/U6 small nuclear ribonucleoprotein Prp4 | Plays role in pre-mRNA splicing as component of the U4/U6-U5 tri-snRNP complex that is involved in spliceosome assembly, and as component of the precatalytic spliceosome (spliceosome B complex). |
| 1.1322798 | sp\|Q14165\|MLEC_HUMAN | Malectin | Carbohydrate-binding protein with a strong ligand preference for Glc2-N-glycan. May play a role in the early steps of protein N-glycosylation. |
| 1.2741292 | sp\|O94925\|GLSK_HUMAN | Glutaminase kidney isoform, mitochondrial | Catalyzes the first reaction in the primary pathway for the renal catabolism of glutamine. Plays a role in maintaining acid-base homeostasis. Regulates the levels of the neurotransmitter glutamate, the main excitatory neurotransmitter in the brain. |
| 1.4723604 | sp\|P15927\|RFA2_HUMAN | Replication protein A 32 kDa subunit | Plays an essential role both in DNA replication and the cellular response to DNA damage. |
| 1.7921993 | sp\|O95251\|KAT7_HUMAN | Histone acetyltransferase KAT7 | Catalytic subunit of histone acetyltransferase HBO1 complexes, which specifically mediate acetylation of histone H3 at 'Lys-14' (H3K14ac), thereby regulating various processes, such as gene transcription, protein ubiquitination, immune regulation, stem cell pluripotent and self-renewal maintenance and embryonic development. |
| 2.1607912 | sp\|Q6DKJ4\|NXN_HUMAN | Nucleoredoxin | Functions as a redox-dependent negative regulator of the Wnt signaling pathway, possibly by preventing ubiquitination of DVL3 by the BCR(KLHL12) complex. |
| 2.1607912 | sp\|O95782\|AP2A1_HUMAN | AP-2 complex subunit alpha-1 | Component of the adaptor protein complex 2 (AP-2). Adaptor protein complexes function in protein transport via transport vesicles in different membrane traffic pathways. |
| 2.7564037 | sp\|P49589\|SYCC_HUMAN | Cysteine--tRNA ligase, cytoplasmic | Catalyzes the ATP-dependent ligation of cysteine to tRNA(Cys). |

**Supplementary Table S6: List of proteins differentially abundant between mutant transfected and non-transfected cells**

| **Fold Change** | **Protein** | **Name** | **Function** |
| --- | --- | --- | --- |
| **Less Abundant** | | | |
| -4.788916 | sp\|P62249\|RS16_HUMAN | 40S ribosomal protein S16 | Structural constituent of ribosome |
| -2.5582538 | sp\|Q9BYD1\|RM13_HUMAN | 39S ribosomal protein L13, mitochondrial | Structural constituent of ribosome |
| -1.3551227 | sp\|Q13505\|MTX1_HUMAN | Metaxin-1 | Involved in transport of proteins into the mitochondrion. |
| -1.1169671 | sp\|Q9Y3B7\|RM11_HUMAN | 39S ribosomal protein L11, mitochondrial | Structural constituent of ribosome |
| -1.0647981 | sp\|Q9Y263\|PLAP_HUMAN | Phospholipase A-2-activating protein | Plays a role in protein ubiquitination, sorting and degradation through its association with VCP. |
| -0.853262 | sp\|P78406\|RAE1L_HUMAN | mRNA export factor | Binds mRNA. May function in nucleocytoplasmic transport and in directly or indirectly attaching cytoplasmic mRNPs to the cytoskeleton. |
| -0.8214491 | sp\|Q9C0B1\|FTO_HUMAN | Alpha-ketoglutarate-dependent dioxygenase FTO | RNA demethylase that mediates oxidative demethylation of different RNA species and acts as a regulator of fat mass, adipogenesis and energy homeostasis. |
| -0.69306874 | sp\|P25705\|ATPA_HUMAN | ATP synthase subunit alpha, mitochondrial | Mitochondrial membrane ATP synthase produces ATP from ADP in the presence of a proton gradient across the membrane which is generated by electron transport complexes of the respiratory chain. |
| -0.63297874 | sp\|P35268\|RL22_HUMAN | 60S ribosomal protein L22 | Structural constituent of ribosome. |
| -0.6043899 | sp\|Q01518\|CAP1_HUMAN | Adenylyl cyclase-associated protein 1 | Directly regulates filament dynamics and has been implicated in a number of complex developmental and morphological processes, including mRNA localization and the establishment of cell polarity. |
| -0.57859886 | sp\|P62826\|RAN_HUMAN | GTP-binding nuclear protein Ran | GTPase involved in nucleocytoplasmic transport, participating both to the import and the export from the nucleus of proteins and RNAs. |
| -0.5629891 | sp\|P00505\|AATM_HUMAN | Aspartate aminotransferase, mitochondrial | Catalyzes the irreversible transamination of the L-tryptophan metabolite L-kynurenine to form kynurenic acid (KA). As a member of the malate-aspartate shuttle, it has a key role in the intracellular NAD(H) redox balance. Is important for metabolite exchange between mitochondria and cytosol, and for amino acid metabolism. |
| -0.5335376 | sp\|P37837\|TALDO_HUMAN | Transaldolase | Important for the balance of metabolites in the pentose-phosphate pathway. |
| -0.46492887 | sp\|P05141\|ADT2_HUMAN | ADP/ATP translocase 2 | ADP:ATP antiporter that mediates import of ADP into the mitochondrial matrix for ATP synthesis, and export of ATP out to fuel the cell. |
| -0.45635405 | sp\|P62913\|RL11_HUMAN | 60S ribosomal protein L11 | Component of the ribosome, a large ribonucleoprotein complex responsible for the synthesis of proteins in the cell. |
| -0.44090268 | sp\|P33993\|MCM7_HUMAN | DNA replication licensing factor MCM7 | Acts as component of the MCM2-7 complex (MCM complex) which is the putative replicative helicase essential for 'once per cell cycle' DNA replication initiation and elongation in eukaryotic cells. |
| -0.4202304 | sp\|P61247\|RS3A_HUMAN | 40S ribosomal protein S3a | Structural constituent of ribosome. |
| -0.3888929 | sp\|P54819\|KAD2_HUMAN | Adenylate kinase 2, mitochondrial | Catalyzes the reversible transfer of the terminal phosphate group between ATP and AMP. Plays an important role in cellular energy homeostasis and in adenine nucleotide metabolism. |
| -0.3642355 | sp\|P50454\|SERPH_HUMAN | Serpin H1 | Binds specifically to collagen. Could be involved as a chaperone in the biosynthetic pathway of collagen. |
| -0.3314769 | sp\|P14618\|KPYM_HUMAN | Pyruvate kinase PKM | Glycolytic enzyme that catalyzes the transfer of a phosphoryl group from phosphoenolpyruvate (PEP) to ADP, generating ATP. |
| -0.32553142 | sp\|P02545\|LMNA_HUMAN | Prelamin-A/C | Lamins are components of the nuclear lamina, a fibrous layer on the nucleoplasmic side of the inner nuclear membrane, which is thought to provide a framework for the nuclear envelope and may also interact with chromatin. |
| -0.29833755 | sp\|P12236\|ADT3_HUMAN | ADP/ATP translocase 3 | ADP:ATP antiporter that mediates import of ADP into the mitochondrial matrix for ATP synthesis, and export of ATP out to fuel the cell. |
| -0.29114786 | sp\|P39019\|RS19_HUMAN | 40S ribosomal protein S19 | Required for pre-rRNA processing and maturation of 40S ribosomal subunits. |
| -0.26152506 | sp\|P50991\|TCPD_HUMAN | T-complex protein 1 subunit delta | Component of the chaperonin-containing T-complex (TRiC), a molecular chaperone complex that assists the folding of proteins upon ATP hydrolysis. |
| -0.25640583 | sp\|P00558\|PGK1_HUMAN | Phopshoglycerate kinase 1 | Catalyzes one of the two ATP producing reactions in the glycolytic pathway via the reversible conversion of 1,3-diphosphoglycerate to 3-phosphoglycerate. |
| -0.2428719 | sp\|P49368\|TCPG_HUMAN | T-complex protein 1 subunit gamma | Component of the chaperonin-containing T-complex (TRiC), a molecular chaperone complex that assists the folding of proteins upon ATP hydrolysis. |
| -0.23416811 | sp\|P15880\|RS2_HUMAN | 40S ribosomal protein S2 | Structural constituent of ribsome. |
| -0.23386714 | sp\|P07741\|APT_HUMAN | Adenine phosphoribosyltransferase | Catalyzes a salvage reaction resulting in the formation of AMP, that is energetically less costly than de novo synthesis. |
| -0.22591352 | sp\|Q15233\|NONO_HUMAN | Non-POU domain-containing octamer-binding protein | DNA- and RNA binding protein, involved in several nuclear processes. |
| -0.19289137 | sp\|P49736\|MCM2_HUMAN | DNA replication licensing factor MCM2 | Acts as component of the MCM2-7 complex (MCM complex) which is the putative replicative helicase essential for 'once per cell cycle' DNA replication initiation and elongation in eukaryotic cells. |
| -0.17863823 | sp\|P04406\|G3P_HUMAN | Glyceraldehyde-3-phosphate dehydrogenase | Has both glyceraldehyde-3-phosphate dehydrogenase and nitrosylase activities, thereby playing a role in glycolysis and nuclear functions, respectively. |
| **More Abundant** | | | |
| 0.3297178 | sp\|P50395\|GDIB_HUMAN | Rab GDP dissociation inhibitor beta | Regulates the GDP/GTP exchange reaction of most Rab proteins by inhibiting the dissociation of GDP from them, and the subsequent binding of GTP to them. |
| 0.3308436 | sp\|Q9NPH2\|INO1_HUMAN | Inositol-3-phosphate synthase 1 | Key enzyme in myo-inositol biosynthesis pathway that catalyzes the conversion of glucose 6-phosphate to 1-myo-inositol 1-phosphate in a NAD-dependent manner. |
| 0.33607486 | sp\|P08133\|ANXA6_HUMAN | Annexin A6 | May associate with CD21. May regulate the release of Ca^2+^ from intracellular stores. |
| 0.34007517 | sp\|P33176\|KINH_HUMAN | Kinesin-1 heavy chain | Microtubule-dependent motor required for normal distribution of mitochondria and lysosomes. |
| 0.40956342 | sp\|P26639\|SYTC_HUMAN | Threonine--tRNA ligase 1, cytoplasmic | Catalyzes the attachment of threonine to tRNA(Thr) in a two-step reaction. |
| 0.5068129 | sp\|P35637\|FUS_HUMAN | RNA binding protein FUS | DNA/RNA-binding protein that plays a role in various cellular processes such as transcription regulation, RNA splicing, RNA transport, DNA repair and damage response. |
| 0.62208825 | sp\|Q06203\|PUR1_HUMAN | Amidophosphoribosyl-transferase | Involved in a subpathway of IMP biosynthesis via de novo pathway, which is itself part of Purine metabolism. |
| 0.71757966 | sp\|P30046\|DOPD_HUMAN | D-dopachrome decarboxylase | Tautomerization of D-dopachrome with decarboxylation to give 5,6-dihydroxyindole (DHI). |
| 0.7993137 | sp\|Q9P0L0\|VAPA_HUMAN | Vesicle-associated membrane protein-associated protein A | May play a role in vesicle trafficking. |
| 1.0460628 | sp\|O15498\|YKT6_HUMAN | Synaptobrevin homolog YKT6 | Vesicular soluble NSF attachment protein receptor mediating vesicle docking and fusion to a specific acceptor cellular compartment. Functions in endoplasmic reticulum to Golgi transport. |
| 1.2047311 | sp\|O94925\|GLSK_HUMAN | Glutaminase kidney isoform, mitochondrial | Catalyzes the first reaction in the primary pathway for the renal catabolism of glutamine. Plays a role in maintaining acid-base homeostasis. Regulates the levels of the neurotransmitter glutamate, the main excitatory neurotransmitter in the brain. |
| 1.2580171 | sp\|P15927\|RFA2_HUMAN | Replication protein A 32 kDa subunit | As part of the heterotrimeric replication protein A complex, binds and stabilizes single-stranded DNA intermediates, that form during DNA replication or upon DNA stress. |
| 1.3545654 | sp\|P43307\|SSRA_HUMAN | Translocon-associated protein subunit alpha | TRAP proteins are part of a complex whose function is to bind calcium to the ER membrane and thereby regulate the retention of ER resident proteins. May be involved in the recycling of the translocation apparatus after completion of the translocation process or may function as a membrane-bound chaperone facilitating folding of translocated proteins. |
| 1.3545654 | sp\|Q9BPX5\|ARP5L_HUMAN | Actin-related protein 2/3 complex subunit 5-like protein | May function as component of the Arp2/3 complex which is involved in regulation of actin polymerization and together with an activating nucleation-promoting factor (NPF) mediates the formation of branched actin networks. |
| 1.3843683 | sp\|Q12874\|SF3A3_HUMAN | Splicing factor 3A subunit 3 | Involved in pre-mRNA splicing as a component of the splicing factor SF3A complex. |
| 1.7049654 | sp\|Q9H3P7\|GCP60_HUMAN | Golgi resident protein GCP60 | Involved in the maintenance of Golgi structure by interacting with giantin, affecting protein transport between the endoplasmic reticulum and Golgi. |
| 1.9275721 | sp\|Q9UPQ0\|LIMC1_HUMAN | LIM and calponin homology domains-containing protein 1 | Actin stress fibers-associated protein that activates non-muscle myosin IIa, negatively regulating cell spreading and cell migration. |
| 2.0022073 | sp\|Q92600\|CNOT9_HUMAN | CCR4-NOT transcription complex subunit 9 | Component of the CCR4-NOT complex which is one of the major cellular mRNA deadenylases and is linked to various cellular processes including bulk mRNA degradation, miRNA-mediated repression, translational repression during translational initiation and general transcription regulation. |
| 2.020335 | sp\|P17096\|HMGA1_HUMAN | High mobility group protein HMG-I/HMG-Y | HMG-I/Y bind preferentially to the minor groove of A+T rich regions in double-stranded DNA. It is suggested that these proteins could function in nucleosome phasing and in the 3'-end processing of mRNA transcripts. They are also involved in the transcription regulation of genes containing, or in close proximity to A+T-rich regions. |
| 2.0204513 | sp\|P61011\|SRP54_HUMAN | Signal recognition particle 54 kDa protein | Binds to the signal sequence of presecretory protein when they emerge from the ribosomes and transfers them to TRAM (translocating chain-associating membrane protein). |
| 2.0311487 | sp\|Q9Y2B0\|CNPY2_HUMAN | Protein canopy homolog 2 | Positive regulator of neurite outgrowth by stabilizing myosin regulatory light chain (MRLC). |
| 2.0645323 | sp\|P63172\|DYLT1_HUMAN | Dynein light chain Tctex-type 1 | Acts as one of several non-catalytic accessory components of the cytoplasmic dynein 1 complex that are thought to be involved in linking dynein to cargos and to adapter proteins that regulate dynein function. Plays a role in neuronal morphogenesis. |
| 2.173147 | sp\|Q12769\|NU160_HUMAN | Nuclear pore complex protein Nup160 | Functions as a component of the nuclear pore complex (NPC). |
| 2.1889472 | sp\|P49840\|GSK3A_HUMAN | Glycogen synthase kinase-3 alpha | Constitutively active protein kinase that acts as a negative regulator in the hormonal control of glucose homeostasis, Wnt signaling and regulation of transcription factors and microtubules. |
| 2.3445802 | sp\|Q15274\|NADC_HUMAN | Nicotinate-nucleotide pyrophosphorylase [carboxylating] | Involved in a subpathway tpart of that is part of the NAD(+) biosynthesis pathway, which is itself part of Cofactor biosynthesis. |
| 2.3445802 | sp\|Q9H223\|EHD4_HUMAN | EH domain-containing protein 4 | ATP- and membrane-binding protein that probably controls membrane reorganization/tubulation upon ATP hydrolysis. Plays a role in early endosomal transport. |
| 2.3649187 | sp\|Q9UGV2\|NDRG3_HUMAN | Protein NDRG3 | Function unclear. May function as a tumour suppressor and may also play important roles in the development of neurological and electrophysiological diseases. |
| 2.3983614 | sp\|Q9HDC9\|APMAP_HUMAN | Adipocyte plasma membrane-associated protein | May play a role in adipocyte differentiation. |
| 2.4703662 | sp\|P29762\|RABP1_HUMAN | Cellular retinoic acid-binding protein 1 | Cytosolic CRABPs may regulate the access of retinoic acid to the nuclear retinoic acid receptors. |
| 2.5580409 | sp\|Q92609\|TBCD5_HUMAN | TBC1 domain family member 5 | May act as a GTPase-activating protein (GAP) for Rab family protein(s). Required for retrograde transport of cargo proteins from endosomes to the trans-Golgi network (TGN). Involved in regulation of autophagy. |

**Supplementary Table S7: List of proteins differentially abundant between mutant transfected and wild-type transfected cells**

| **Fold Change** | **Protein** | **Name** | **Function** |
| --- | --- | --- | --- |
| **Less Abundant** | | | |
| -2.4344592 | sp\|O95782\|AP2A1_HUMAN | AP-2 complex subunit alpha-1 | Component of the adaptor protein complex 2 (AP-2). Adaptor protein complexes function in protein transport via transport vesicles in different membrane traffic pathways. |
| -1.7803267 | sp\|P30876\|RPB2_HUMAN | DNA-directed RNA polymerase II subunit RPB2 | DNA-dependent RNA polymerase catalyzes the transcription of DNA into RNA using the four ribonucleoside triphosphates as substrates. |
| -1.5180521 | sp\|P52788\|SPSY_HUMAN | Spermine synthase | Catalyzes the production of spermine from spermidine and decarboxylated S-adenosylmethionine (dcSAM). |
| -1.335764 | sp\|Q9BTY7\|HGH1_HUMAN | Protein HGH1 homolog | Chaperone-mediated protein folding |
| -1.0895904 | sp\|P60763\|RAC3_HUMAN | Ras-related C3 botulinum toxin substrate 3 | Plasma membrane-associated small GTPase. In its active state binds to a variety of effector proteins to regulate cellular responses, such as cell spreading and the formation of actin-based protrusions including lamellipodia and membrane ruffles. |
| -1.0788805 | sp\|Q7L1Q6\|BZW1_HUMAN | Basic leucine zipper and W2 domain-containing protein 1 | Enhances histone H4 gene transcription but does not seem to bind DNA directly. |
| -1.0704813 | sp\|O43172\|PRP4_HUMAN | U4/U6 small nuclear ribonucleoprotein Prp4 | Plays role in pre-mRNA splicing as component of the U4/U6-U5 tri-snRNP complex that is involved in spliceosome assembly, and as component of the precatalytic spliceosome (spliceosome B complex). |
| -0.79752356 | sp\|Q05193\|DYN1_HUMAN | Dynamin-1 | Microtubule-associated force-producing protein involved in producing microtubule bundles and able to bind and hydrolyze GTP. Most probably involved in vesicular trafficking processes. Involved in receptor-mediated endocytosis. |
| -0.62357765 | sp\|P21912\|SDHB_HUMAN | Succinate dehydrogenase [ubiquinone] iron-sulfur subunit, mitochondrial | Iron-sulfur protein (IP) subunit of succinate dehydrogenase (SDH) that is involved in complex II of the mitochondrial electron transport chain and is responsible for transferring electrons from succinate to ubiquinone (coenzyme Q). |
| -0.5995171 | sp\|P36871\|PGM1_HUMAN | Phosphoglucomutase-1 | This enzyme participates in both the breakdown and synthesis of glucose. |
| -0.45797586 | sp\|P35659\|DEK_HUMAN | Protein DEK | Involved in chromatin organization. |
| -0.41750172 | sp\|P62873\|GBB1_HUMAN | Guanine nucleotide-binding protein G(I)/G(S)/G(T) subunit beta-1 | Guanine nucleotide-binding proteins (G proteins) are involved as a modulator or transducer in various transmembrane signaling systems. The beta and gamma chains are required for the GTPase activity, for replacement of GDP by GTP, and for G protein-effector interaction. |
| -0.34489024 | sp\|P31939\|PUR9_HUMAN | Bifunctional purine biosynthesis protein ATIC | Bifunctional enzyme that catalyzes the last two steps of purine biosynthesis. |
| -0.2783318 | sp\|P11177\|ODPB_HUMAN | Pyruvate dehydrogenase E1 component subunit beta, mitochondrial | The pyruvate dehydrogenase complex catalyzes the overall conversion of pyruvate to acetyl-CoA and CO_2_, and thereby links the glycolytic pathway to the tricarboxylic cycle. |
| -0.24159154 | sp\|P49368\|TCPG_HUMAN | T-complex protein 1 subunit gamma | Component of the chaperonin-containing T-complex (TRiC), a molecular chaperone complex that assists the folding of proteins upon ATP hydrolysis. |
| -0.13958682 | sp\|Q04837\|SSBP_HUMAN | Single-stranded DNA-binding protein, mitochondrial | In vitro, required to maintain the copy number of mitochondrial DNA (mtDNA) and plays crucial roles during mtDNA replication that stimulate activity of the replisome components POLG and TWNK at the replication fork. |
| **More Abundant** | | | |
| 0.29401335 | sp\|P16104\|H2AX_HUMAN | Histone H2AX | Variant histone H2A which replaces conventional H2A in a subset of nucleosomes. Histones thereby play a central role in transcription regulation, DNA repair, DNA replication and chromosomal stability. |
| 0.3704586 | sp\|P63220\|RS21_HUMAN | 40S ribosomal protein S21 | Structural constituent of ribosome. |
| 0.38168684 | sp\|P55060\|XPO2_HUMAN | Exportin-2 | Export receptor for importin-alpha. Mediates importin-alpha re-export from the nucleus to the cytoplasm after import substrates (cargos) have been released into the nucleoplasm. |
| 0.43802047 | sp\|P62140\|PP1B_HUMAN | Serine/threonine-protein phosphatase PP1-beta catalytic subunit | Protein phosphatase that associates with over 200 regulatory proteins to form highly specific holoenzymes which dephosphorylate hundreds of biological targets. Protein phosphatase (PP1) is essential for cell division, it participates in the regulation of glycogen metabolism, muscle contractility and protein synthesis. Involved in regulation of ionic conductance and long-term synaptic plasticity. |
| 0.5287636 | sp\|P53999\|TCP4_HUMAN | Activated RNA polymerase II transcriptional coactivator p15 | General coactivator that functions cooperatively with TAFs and mediates functional interactions between upstream activators and the general transcriptional machinery. May be involved in stabilizing the multiprotein transcription complex. |
| 0.53964293 | sp\|P38159\|RBMX_HUMAN | RNA-binding motif protein, X chromosome | RNA-binding protein that plays several role in the regulation of pre- and post-transcriptional processes. Implicated in tissue-specific regulation of gene transcription and alternative splicing of several pre-mRNAs. |
| 0.63821447 | sp\|P35637\|FUS_HUMAN | RNA-binding protein FUS | DNA/RNA-binding protein that plays a role in various cellular processes such as transcription regulation, RNA splicing, RNA transport, DNA repair and damage response. |
| 0.82862306 | sp\|Q9P0L0\|VAPA_HUMAN | Vesicle-associated membrane protein-associated protein A | May play a role in vesicle trafficking. |
| 0.8773641 | sp\|P30046\|DOPD_HUMAN | D-dopachrome decarboxylase | Tautomerization of D-dopachrome with decarboxylation to give 5,6-dihydroxyindole (DHI). |
| 0.9104095 | sp\|Q9H814\|PHAX_HUMAN | Phosphorylated adapter RNA export protein | A phosphoprotein adapter involved in the XPO1-mediated U snRNA export from the nucleus. |
| 0.9281879 | sp\|Q15637\|SF01_HUMAN | Splicing factor 1 | Necessary for the ATP-dependent first step of spliceosome assembly. |
| 0.96657825 | sp\|P35527\|K1C9_HUMAN | Keratin, type I cytoskeletal 9 | Plays a role in keratin filament assembly. |
| 1.0312784 | sp\|Q9H307\|PININ_HUMAN | Pinin | Transcriptional activator binding to the E-box 1 core sequence of the E-cadherin promoter gene. |
| 1.2384619 | sp\|P98179\|RBM3_HUMAN | RNA-binding protein 3 | Cold-inducible mRNA binding protein that enhances global protein synthesis at both physiological and mild hypothermic temperatures. |
| 1.2384619 | sp\|P62851\|RS25_HUMAN | 40S ribosomal protein S25 | Structural constituent of ribosome. |
| 1.3642771 | sp\|P62753\|RS6_HUMAN | 40S ribosomal protein S6 | Component of the 40S small ribosomal subunit. |
| 1.4416274 | sp\|P62917\|RL8_HUMAN | 60S ribosomal protein L8 | Component of the large ribosomal subunit. |
| 1.8834004 | sp\|P51784\|UBP11_HUMAN | Ubiquitin carboxyl-terminal hydrolase 11 | Protease that can remove conjugated ubiquitin from target proteins and polyubiquitin chains. Inhibits the degradation of target proteins by the proteasome. |
| 2.2395134 | sp\|P07858\|CATB_HUMAN | Cathepsin B | Thiol protease which is believed to participate in intracellular degradation and turnover of proteins. |
| 2.3365922 | sp\|Q8ND24\|RN214_HUMAN | RING finger protein 214 | Involved in protein ubiquitination. |
| 2.888747 | sp\|P17096\|HMGA1_HUMAN | High mobility group protein HMG-I/HMG-Y | HMG-I/Y bind preferentially to the minor groove of A+T rich regions in double-stranded DNA. It is suggested that these proteins could function in nucleosome phasing and in the 3'-end processing of mRNA transcripts. They are also involved in the transcription regulation of genes containing, or in close proximity to A+T-rich regions. |

**Supplementary Table S8: List of proteins differentially abundant between empty vector transfected and non-transfected cells**

| **Fold Change** | **Protein** | **Name** | **Function** |
| --- | --- | --- | --- |
| **Less Abundant** | | | |
| -2.650073 | sp\|O14949\|QCR8_HUMAN | Cytochrome b-c1 complex subunit 8 | Component of the ubiquinol-cytochrome c oxidoreductase, a multisubunit transmembrane complex that is part of the mitochondrial electron transport chain which drives oxidative phosphorylation. |
| -2.5518029 | sp\|Q10713\|MPPA_HUMAN | Mitochondrial-processing peptidase subunit alpha | Substrate recognition and binding subunit of the essential mitochondrial processing protease (MPP), which cleaves the mitochondrial sequence off newly imported precursors proteins. |
| -1.7825811 | sp\|P24928\|RPB1_HUMAN | DNA-directed RNA polymerase II subunit RPB1 | DNA-dependent RNA polymerase catalyzes the transcription of DNA into RNA using the four ribonucleoside triphosphates as substrates. Largest and catalytic component of RNA polymerase II which synthesizes mRNA precursors and many functional non-coding RNAs. |
| -1.4472072 | sp\|Q9C0B1\|FTO_HUMAN | Alpha-ketoglutarate-dependent dioxygenase FTO | RNA demethylase that mediates oxidative demethylation of different RNA species, such as mRNAs, tRNAs and snRNAs, and acts as a regulator of fat mass, adipogenesis and energy homeostasis. |
| -0.8712561 | sp\|Q15149\|PLEC_HUMAN | Plectin | Interlinks intermediate filaments with microtubules and microfilaments and anchors intermediate filaments to desmosomes or hemidesmosomes. |
| -0.8128491 | sp\|P13861\|KAP2_HUMAN | cAMP-dependent protein kinase type II-alpha regulatory subunit | Regulatory subunit of the cAMP-dependent protein kinases involved in cAMP signaling in cells. |
| -0.5686383 | sp\|P53004\|BIEA_HUMAN | Biliverdin reductase A | Reduces the gamma-methene bridge of the open tetrapyrrole, biliverdin IX alpha, to bilirubin with the concomitant oxidation of a NADH or NADPH cofactor. |
| -0.49464598 | sp\|Q92499\|DDX1_HUMAN | ATP-dependent RNA helicase DDX1 | Acts as an ATP-dependent RNA helicase, able to unwind both RNA-RNA and RNA-DNA duplexes. |
| **More Abundant** | | | |
| 0.2042159 | sp\|P06733\|ENOA_HUMAN | Alpha enolase | Glycolytic enzyme the catalyzes the conversion of 2-phosphoglycerate to phosphoenolpyruvate |
| 0.48937234 | sp\|P63279\|UBC9_HUMAN | SUMO-conjugating enzyme UBC9 | Involved in the pathway protein sumoylation, which is part of Protein modification |
| 0.4961204 | sp\|P55010\|IF5_HUMAN | Eukaryotic translation initiation factor 5 | Catalyzes the hydrolysis of GTP bound to the 40S ribosomal initiation complex with the subsequent joining of a 60S ribosomal subunit. |
| 0.509079 | sp\|Q9BY32\|ITPA_HUMAN | Inosine triphosphate pyrophosphatase | Pyrophosphatase that hydrolyzes the non-canonical purine nucleotides to their respective monophosphate derivatives. |
| 0.56471723 | sp\|P30049 \|ATPD_HUMAN | ATP synthase subunit delta, mitochondrial | Mitochondrial membrane ATP synthase produces ATP from ADP in the presence of a proton gradient across the membrane which is generated by electron transport complexes of the respiratory chain. |
| 0.5949262 | sp\|P61160\|ARP2_HUMAN | Actin-related protein 2 | ATP-binding component of the Arp2/3 complex, a multiprotein complex that mediates actin polymerization upon stimulation by nucleation-promoting factor (NPF). |
| 0.6654975 | sp\|P68871\|HBB_HUMAN | Hemoglobin subunit beta | Involved in oxygen transport from the lung to the various peripheral tissues. |
| 0.77291125 | sp\|P35250\|RFC2_HUMAN | Replication factor C subunit 2 | The elongation of primed DNA templates by DNA polymerase delta and epsilon requires the action of the accessory proteins proliferating cell nuclear antigen (PCNA) and activator 1. This subunit binds ATP. |
| 0.9915015 | sp\|P01008\|ANT3_HUMAN | Antithrombin-III | Most important serine protease inhibitor in plasma that regulates the blood coagulation cascade. |
| 1.1482236 | sp\|P37840\|SYUA_HUMAN | Alpha-synuclein | Neuronal protein that plays several roles in synaptic activity such as regulation of synaptic vesicle trafficking and subsequent neurotransmitter release. |
| 1.4167656 | sp\|O00303\|EIF3F_HUMAN | Eukaryotic translation initiation factor 3 subunit F | Component of the eukaryotic translation initiation factor 3 (eIF-3) complex, which is required for several steps in the initiation of protein synthesis. |
| 1.5193136 | sp\|Q15773\|MLF2_HUMAN | Myeloid leukemia factor 2 | Regulation of transcription. |
| 1.5193136 | sp\|P61086\|UBE2K_HUMAN | Ubiquitin-conjugating enzyme E2 K | Accepts ubiquitin from the E1 complex and catalyzes its covalent attachment to other proteins. |
| 1.9315825 | sp\|P29762\|RABP1_HUMAN | Cellular retinoic acid-binding protein 1 | May regulate the access of retinoic acid to the nuclear retinoic acid receptors. |
| 2.3856065 | sp\|Q92600\|CNOT9_HUMAN | CCR4-NOT transcription complex subunit 9 | Component of the CCR4-NOT complex which is one of the major cellular mRNA deadenylases and is linked to various cellular processes including bulk mRNA degradation, miRNA-mediated repression, translational repression during translational initiation and general transcription regulation. |
| 2.527652 | sp\|Q16864\|VATF_HUMAN | V-type proton ATPase subunit F | Subunit of the peripheral V1 complex of vacuolar ATPase essential for assembly or catalytic function. V-ATPase is responsible for acidifying a variety of intracellular compartments in eukaryotic cells. |
| 2.527652 | sp\|P49753\|ACOT2_HUMAN | Acyl-coenzyme A thioesterase 2, mitochondrial | Acyl-CoA thioesterases are a group of enzymes that catalyze the hydrolysis of acyl-CoAs to the free fatty acid and coenzyme A (CoASH), providing the potential to regulate intracellular levels of acyl-CoAs, free fatty acids and CoASH. The enzyme is involved in enhancing the hepatic fatty acid oxidation in mitochondria. |
| 3.0763242 | sp\|P19525\|E2AK2_HUMAN | Interferon-induced, double-stranded RNA-activated protein kinase | IFN-induced dsRNA-dependent serine/threonine-protein kinase that phosphorylates the alpha subunit of eukaryotic translation initiation factor 2 (EIF2S1/eIF-2-alpha) and plays a key role in the innate immune response to viral infection. |
| 3.1480145 | sp\|Q9P013\|CWC15_HUMAN | Spliceosome-associated protein CWC15 homolog | Involved in pre-mRNA splicing as component of the spliceosome. |
| 3.4633446 | sp\|Q9H910\|JUPI2_HUMAN | Jupiter microtubule associated homolog 2 | Nicotinic Acid Adenine Dinucleotide Phosphate (NAADP) binding protein required for NAADP-evoked intracellular calcium release. |
